# Surface defect detection method for shawo radishes based on improved RT-DETR

**DOI:** 10.3389/fnut.2026.1890294

**Published:** 2026-08-03

**Authors:** Liying Ma, Xufeng Hua, Na Zhang, Xiaoxue Li, Chunyang Qian, Cunkun Chen, Chenghu Dong, Zhiqiang Zhu, Lihong Hao

**Affiliations:** 1 College of Computer and Information Engineering, Tianjin Agricultural University, Tianjin, China; 2 State Key Laboratory of Vegetable Biobreeding, Key Laboratory of Storage and Preservation of Agricultural Products, Tianjin Key Laboratory of Postharvest Physiology and Storage and Preservation of Agricultural Products, Institute of Agricultural Products Preservation and Processing Technology (National Engineering and Technology Research Center for Preservation of Agricultural Products), Tianjin Academy of Agricultural Sciences, Ministry of Agriculture and Rural Affairs, Tianjin, China; 3 Huangnan Tibetan Autonomous Prefecture Agriculture and Animal Husbandry Comprehensive Service Center, Tongren, Qinghai, China; 4 Tianjin Xiaowo Village Guli Fruit and Vegetable Planting Farmers' Professional Cooperative, Tianjin, China

**Keywords:** computer vision, deep learning, RT-DETR, shawo radish, surface defects, wavelet transform

## Abstract

**Introduction:**

Surface defect detection of shawo radishes is challenged by complex defect characteristics, large variations in defect scale, and insufficient feature representation, which can limit automated quality inspection and grading performance.

**Methods:**

To address these challenges, this study proposes an improved RT-DETR-r18 framework based on a frequency–spatial collaborative modeling strategy. The proposed model incorporates a Wavelet Transform Block (WT_Block), an Attention-based Intra-scale Feature Interaction with High-Low Attention module (AIFI-HiLo), and a VoVGSCSP-PConv Cross-Scale Feature Fusion Module (VP_CCFM) to enhance feature extraction, feature interaction, and multi-scale representation.

**Results:**

Experiments conducted on a self-constructed shawo radish surface defect dataset showed that the proposed method achieved a Precision of 94.5%, a Recall of 81.2%, and a mean Average Precision at an Intersection over Union threshold of 0.5 (mAP@50) of 85.3%, representing improvements of 2.9, 2.8, and 1.9 percentage points over the baseline RT-DETR-r18 model, respectively. Meanwhile, the parameter count and computational complexity were reduced by 7 and 12.7%, respectively. Comparative experiments further showed that the proposed model outperformed several mainstream object detection methods in overall detection performance.

**Discussion:**

These results indicate that the proposed frequency–spatial collaborative framework can improve the accuracy, robustness, and efficiency of shawo radish surface defect detection, providing technical support for automated quality inspection and intelligent grading of root vegetable products.

## 1 Introduction

Shawo radish, a specialty agricultural product from Xiqing District, Tianjin, is rich in anthocyanins, vitamin C, and other nutrients, making it highly valued in premium consumer and gift markets (1). During postharvest handling and quality grading, the external appearance of agricultural products directly influences product classification and market price, underscoring the necessity for accurate and efficient quality assessment. Although non-destructive testing techniques, particularly visible/near-infrared (Vis/NIR) spectroscopy combined with machine learning, have been widely applied to internal constituent analysis as well as authentication and quality evaluation of high-value agricultural products (2), these approaches primarily depend on spectral information and are generally ineffective in detecting surface defects that require spatial geometric information. Surface appearance is a key indicator of commercial quality, and even minor surface defects can reduce commercial value and consumer acceptance. Therefore, efficient and accurate surface defect detection is essential for maintaining the market competitiveness of shawo radish products.

Traditional fruit and vegetable defect detection mainly relies on manual inspection or machine vision methods based on handcrafted features, which often suffer from low efficiency, strong subjectivity, and limited robustness (3). With the rapid development of deep learning, object detection approaches have become the dominant technical solution (4, 5). Among them, two-stage detectors represented by the R-CNN series achieve high detection accuracy through region proposal generation and refined classification (6–8). However, their high computational complexity makes them difficult to deploy in real-time high-speed sorting scenarios. In automated sorting systems, overall latency is determined by both the inference time of the detection network and the image-acquisition processes, including optical focusing, zoom adjustment, and operational delays under varying illumination conditions (9). From a system-level perspective, these factors collectively constrain the real-time performance of the entire processing pipeline. In contrast, one-stage detectors represented by YOLO perform object localization and classification in an end-to-end manner, significantly reducing computational overhead and improving overall processing efficiency at the algorithm level (10–12). Nevertheless, CNN-based one-stage models are constrained by limited receptive fields, which make it difficult to capture long-range dependencies, particularly in scenarios involving complex textures and multi-scale defects (13). In addition, their reliance on Non-Maximum Suppression (NMS) for post-processing may lead to missed or false detections in densely distributed or adjacent targets and introduce an inference bottleneck (14, 15).

To address the limitations of CNN architectures, researchers have introduced Transformer-based methods into object detection tasks (16). DETR formulates object detection as a set prediction problem and employs an encoder–decoder architecture to achieve end-to-end detection without relying on NMS (17). However, DETR suffers from slow convergence and limited performance on small objects. To overcome these issues, Deformable DETR introduces deformable attention mechanisms, reducing computational complexity while improving small-object detection performance (18). Building upon this framework, RT-DETR further integrates global attention and multi-scale feature modeling, achieving a favorable balance between accuracy and efficiency for real-time detection tasks (19–21). Despite its strong performance in complex environments, RT-DETR still has several limitations. Its ResNet-based backbone relies on fixed-scale convolutions, making it difficult to represent the frequency characteristics of objects at different scales within a unified feature space. Moreover, inconsistencies in semantic hierarchies and insufficient cross-scale interactions during feature fusion can lead to the loss of critical information. To address cross-layer semantic gaps under lightweight constraints, recent machine vision paradigms frequently employ multi-branch Feature Pyramid Networks (FPN) and adaptive upsampling modules to enhance hierarchical information flow and spatial consistency. These strategies offer valuable guidance for optimizing multi-scale representations (37).

In the task of shawo radish surface defect detection, defects exhibit substantial variations not only in spatial scale but also in representation characteristics. Small-scale defects typically manifest as local, high-frequency texture disturbances with low contrast and low signal-to-noise ratios, making them easily confused with background textures. In contrast, large-scale defects primarily manifest as structural deformations, which are identified using low-frequency contour information and global contextual relationships. Furthermore, the dense distribution of small defects increases the risk of missed detections.

To address the limitations of existing methods in modeling multi-scale defects, particularly the insufficient characterization of frequency features and the limited interaction between cross-scale information, this study proposes an improved RT-DETR-r18-based framework for detecting surface defects on shawo radish. A frequency–spatial collaborative modeling framework is developed to optimize the network from three aspects: frequency-domain feature representation, frequency-decoupled attention mechanisms, and cross-scale feature fusion. The proposed approach aims to improve the detection accuracy of multi-scale surface defects and densely distributed small targets, thereby providing technical support for shawo radish quality grading.

## 2 Improved RT-DETR model

The RT-DETR model achieves a favorable balance between detection accuracy and inference speed. Its overall architecture consists of four main components: a backbone network, an Efficient Hybrid Encoder, a Transformer decoder, and a prediction head (22). The backbone network extracts multi-level feature representations; the hybrid encoder enhances and cross-scale-fuses features; the decoder performs object localization and classification via a query-based mechanism; and the prediction head generates the final detection results.

During the encoding stage, RT-DETR employs an efficient hybrid encoder consisting of Attention-based Intra-scale Feature Interaction (AIFI) and CNN-based Cross-scale Feature Fusion. AIFI performs intra-scale interaction on the high-level S5 feature map using a single-scale Transformer encoder, whereas the cross-scale fusion module integrates features across multiple resolutions.

**Figure 1:**
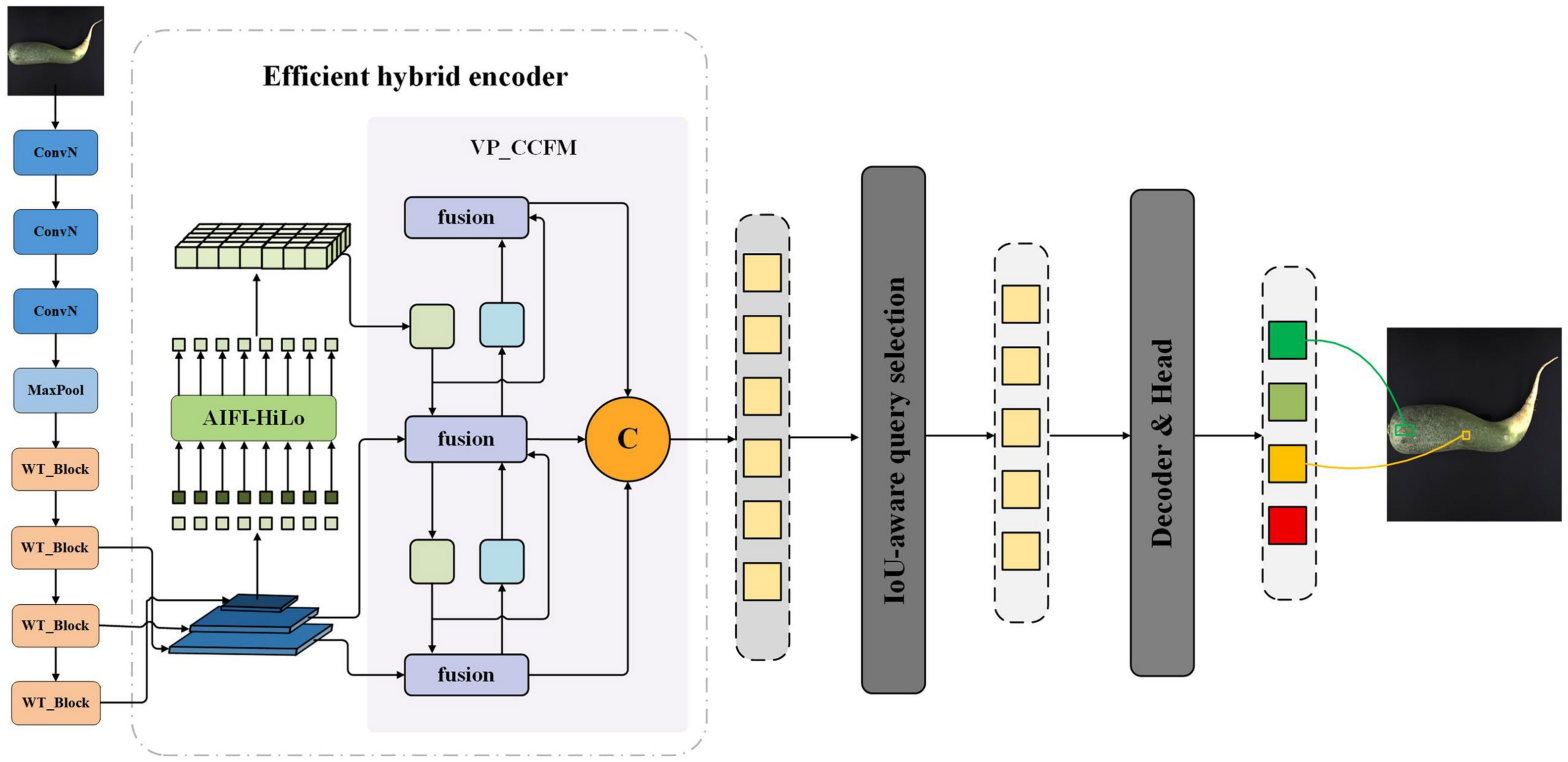
Architecture of the improved RT-DETR model.

RT-DETR-r18 was selected as the baseline because it is a lightweight variant of the RT-DETR framework with fewer parameters and lower computational cost than larger variants such as RT-DETR-r50 and RT-DETR-L. Since the objective of this study is to evaluate whether the proposed modules can improve the detection capability of a compact model, RT-DETR-r18 provides a more suitable baseline. Based on this architecture, three targeted improvements are introduced to enhance multi-scale feature representation, frequency-aware feature modeling, and feature fusion efficiency. First, a wavelet convolution-based WT_Block (Wavelet Transform Block) replaces the original BasicBlock in the backbone network. This module performs multi-scale frequency-domain decomposition, jointly representing high-frequency details and low-frequency information to better capture multi-scale defects (23). Second, the HiLo attention mechanism is added to form the AIFI-HiLo (Attention-based Intra-scale Feature Interaction with High-Low Attention) module. By separating high- and low-frequency features, the module models local details and global contextual information independently, thereby improving small-defect detection (24). Finally, a VP_CCFM (VoVGSCSP-PConv Cross-Scale Feature Fusion Module) enhances feature fusion by replacing RepC3 (Re-parameterized C3) with VoVGSCSP (VoVNet GSConv Cross Stage Partial), thereby boosting cross-level feature reuse and multi-scale fusion (25). Partial Convolution (PConv) is also adopted for selective channel computation, lowering computational cost without reducing representational power (26). The improved network architecture is shown in Figure 1.

### 2.1 WT_Block module

The discriminative information of shawo radish surface defects exhibits a multi-resolution distribution in the frequency domain, where structures at different scales correspond to distinct frequency-response patterns. Therefore, effective defect representation requires a mechanism capable of separating and reconstructing different frequency components. However, the BasicBlock in the conventional ResNet-18 backbone mainly relies on fixed-scale convolution kernels, which limits its ability to explicitly model multi-resolution frequency information and may restrict the representation of complex defect structures (27). Wavelet transform has been increasingly introduced into deep visual models because it can decompose feature maps into low- and high-frequency components while retaining spatial–frequency information. Fan et al. (28) proposed a wavelet-based multi-level information compensation learning method for visible-infrared person re-identification, in which wavelet decomposition was used to preserve discriminative details and enhance local information. Inspired by this strategy, this study introduces WT_Block based on the two-dimensional Haar wavelet transform to strengthen texture discontinuities, boundary information, and fine-grained defect cues in shawo radish surface images.

The proposed module uses orthogonal wavelet bases to decompose input features into low-frequency approximation components and high-frequency detail components. This enables explicit scale separation in the frequency domain. With its joint spatial–frequency localization capability, the wavelet transform can capture subtle local defects while preserving global structural consistency. This enhances the representation of defect features with diverse scales, textures, and structural characteristics. Most convolutional neural networks address scale variation through multi-scale architectural designs. However, their multi-scale representations mainly rely on implicit feature learning in deep networks and lack explicit modeling and decoupling of frequency components. In contrast, wavelet transforms use the rigorous theory of Multi-Resolution Analysis (MRA). This enables hierarchical decomposition of features across different scales and frequency bands within a unified framework. This approach facilitates the hierarchical representation of features across multiple scales and frequency bands, thereby providing complementary frequency-domain information for subsequent feature fusion and attention modeling.

**Figure 2:**
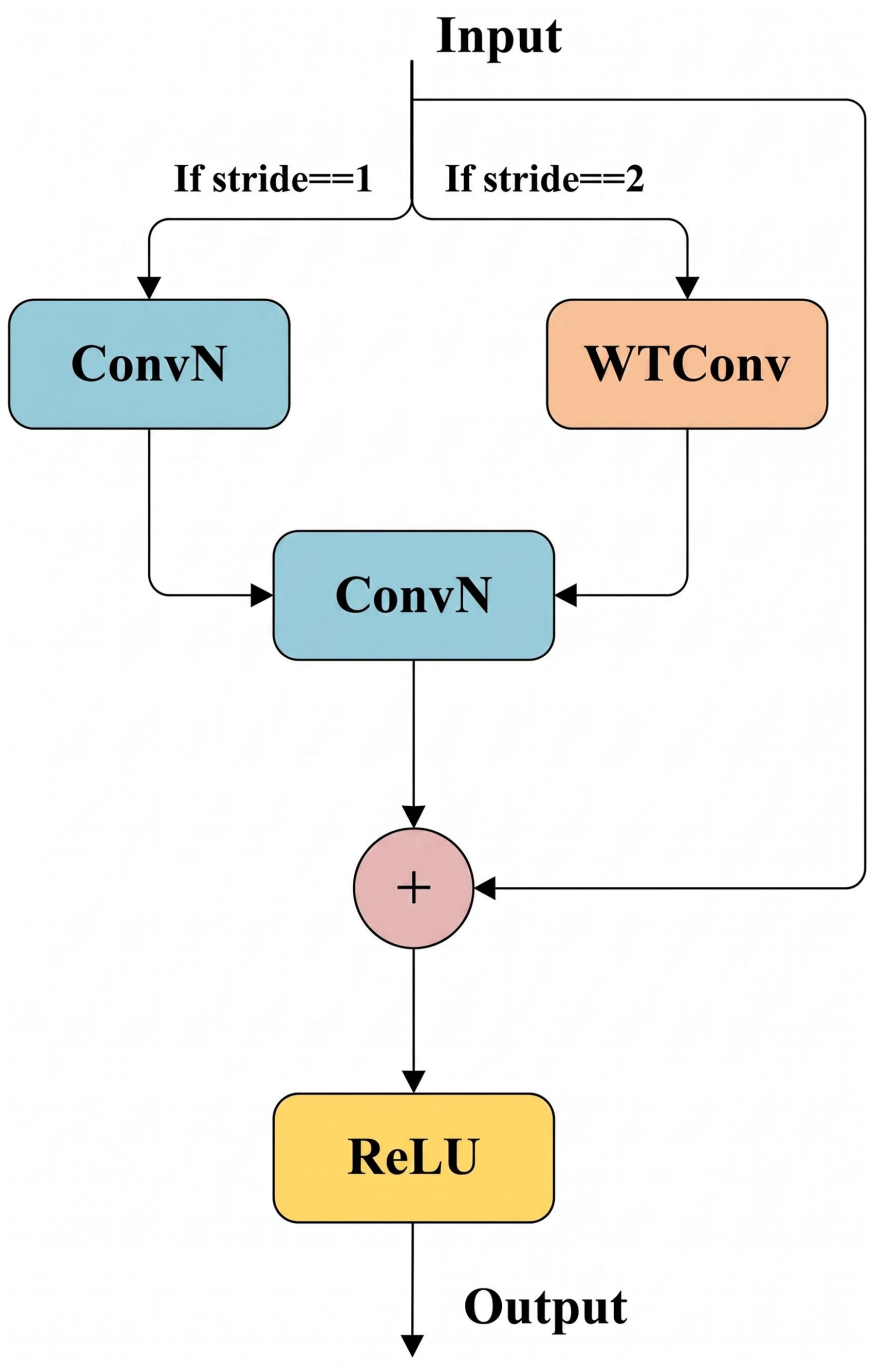
Structure of the WT_Block module.

WT_Block uses a differentiated architecture design strategy. When the stride is set to 2, wavelet convolution is applied for multi-scale decomposition and to expand the receptive field. When the stride is set to 1, standard convolution is used to preserve spatial resolution and local structural information. This hybrid approach combines frequency-domain enhancement with spatial information preservation. It improves multi-scale feature representation and effectively controls computational complexity. As a result, the network achieves a balance between accuracy and efficiency. The WT_Block design is shown in Figure 2.

**Figure 3:**
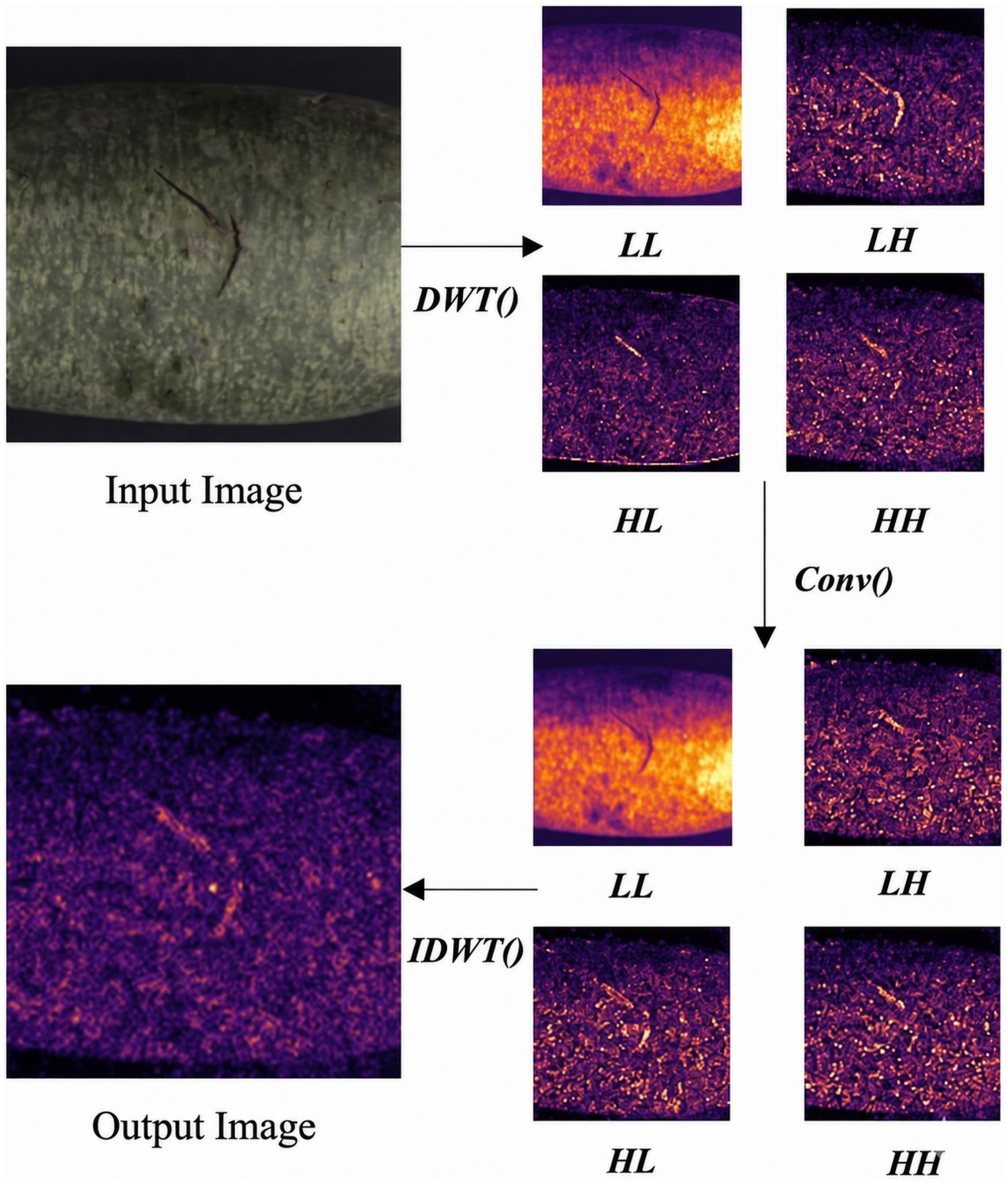
Pseudo-color visualization of the low-frequency (LL) and high-frequency (LH, HL, and HH) sub-band features produced by the DWT operation in WT_Block, together with the reconstructed output after convolution and IDWT.

Within the wavelet convolution module, the input feature map is decomposed by the Haar Discrete Wavelet Transform (DWT) into four frequency sub-bands denoted as $X_{\mathrm{LL}}$ , $X_{\mathrm{LH}}$ , $X_{\mathrm{HL}}$ , and $X_{\mathrm{HH}}$ , respectively. Among these sub-bands, the low-frequency component is downsampled to compress global contextual information, and its receptive field is progressively enlarged in subsequent network layers to capture the overall structural characteristics of the target. In contrast, the high-frequency sub-bands preserve directional detail information such as edges, textures, and local discontinuities. They are mainly responsible for extracting directional local detail features. Together, they form a parallel multi-scale feature representation framework. Subsequently, the four sub-bands are processed by independent convolutional layers to enhance features and reconstruct information. The enhanced features are then concatenated and restored to the original spatial resolution through the Inverse Discrete Wavelet Transform (IDWT), enabling effective fusion of multi-frequency information (29). To intuitively illustrate the frequency-domain decomposition and reconstruction process within WT_Block, the low-frequency sub-band (LL) and three high-frequency sub-bands (LH, HL, and HH) generated by the DWT operation are visualized in pseudo-color, together with the reconstructed feature representation after convolution and IDWT, as shown in Figure 3. The wavelet-domain convolution operation at the i -th decomposition level is formulated in Equation 1.


$$X_{\text{out}}^{i}=\text{Conv}(W_{\text{base}},X^{i-1})+\text{IDWT}(\text{Conv}(W^{i},\mathrm{DWT}(X_{\mathrm{LL}}^{i-1})))$$ (1)

where $X_{\text{out}}^{i}$ denotes the output feature map of the i -th layer; $W_{\text{base}}$ represents the weights of the base convolution; $X^{i-1}$ denotes the feature map from the (i−1) -th layer; IDWT() denotes the inverse wavelet transform operation; $W^{i}$ represents the sub-band convolution weights in the wavelet domain at the i -th decomposition level; DWT() denotes the two-dimensional Haar wavelet transform; and $X_{\mathrm{LL}}^{i-1}$ denotes the low-frequency approximation component at the (i−1) -th decomposition level.

### 2.2 AIFI-HiLo module

For shawo radish surface defect detection, defect regions often exhibit complex visual characteristics, including weak texture contrast, irregular morphology, and local boundary discontinuities. These characteristics require the model to simultaneously capture local fine-grained defect details and global contextual information. Previous studies have shown that multi-granularity feature interaction can improve representation robustness by integrating global semantic information with local discriminative details. Chen et al. (30) further demonstrated that explicit alignment between global and local features helps maintain semantic consistency and enhance feature robustness. Motivated by this global–local complementary modeling concept, AIFI-HiLo is introduced in this study to strengthen the interaction between high-frequency local details and low-frequency global semantic information, thereby improving defect-related feature selection in the encoder.

In the baseline RT-DETR model, the AIFI module adopts a Transformer-based structure consisting of multi-head self-attention (MSA) and feed-forward networks (FFN) for feature extraction. MSA establishes long-range dependencies among different spatial locations and effectively captures global contextual information. However, conventional self-attention mechanisms treat feature components in a relatively uniform manner and do not explicitly distinguish between different frequency characteristics. As a result, fine-grained local details may be weakened during global feature aggregation, which limits the representation of complex defect patterns with diverse scales and textures (31).

From a frequency-domain perspective, self-attention can be regarded as an adaptive global information aggregation mechanism, which tends to emphasize low-frequency structural components related to global semantic patterns. In contrast, high-frequency components, which contain fine-grained details such as edges, textures, and local defect characteristics, may receive insufficient attention during feature aggregation. To alleviate this limitation, the HiLo attention mechanism is embedded into the AIFI module to construct the proposed AIFI-HiLo module. By separating high-frequency local detail modeling from low-frequency global context modeling, AIFI-HiLo enables collaborative representation of local defect cues and global contextual information, thereby providing complementary spatial–frequency features for defect representation learning.

**Figure 4:**
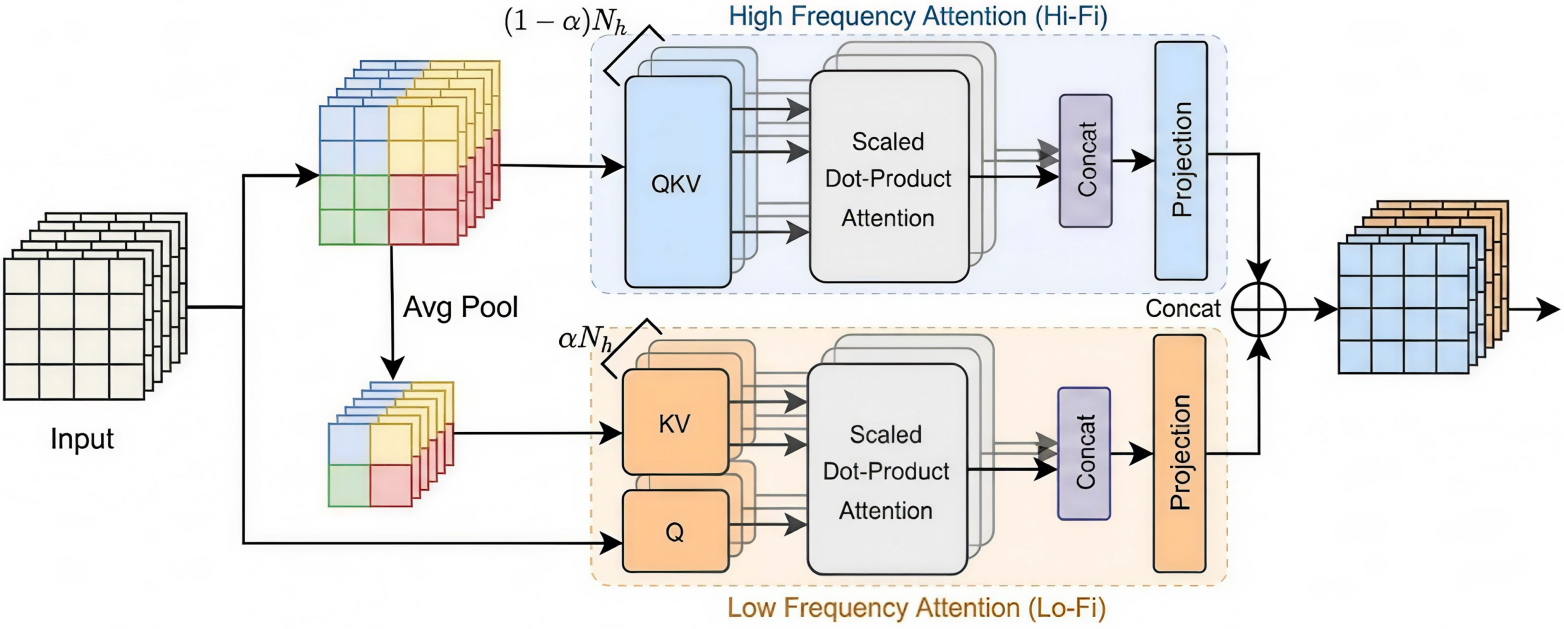
Schematic diagram of the HiLo high–low frequency separation mechanism.

Specifically, the HiLo attention mechanism adopts a dual-branch architecture. The high-frequency branch (Hi-Fi) preserves the original feature map resolution and employs local-window attention to enhance fine-grained feature representation. In contrast, the low-frequency branch (Lo-Fi) first downsamples the feature map and then applies global self-attention to capture overall structural information. The outputs of the two branches are fused through channel concatenation, followed by a 1 × 1 convolution, enabling a collaborative representation of local details and global contextual information. The architecture of the module is illustrated in Figure 4 (24).

Compared with conventional multi-head self-attention, the proposed AIFI-HiLo module explicitly models high- and low-frequency features, enabling the network to simultaneously capture local fine-grained details and global contextual information. By constraining the attention range of the high-frequency branch while preserving global perception via the low-frequency branch, the module enhances feature discrimination and reduces interference from complex backgrounds. After embedding the HiLo mechanism into the AIFI module, the network achieves more effective global–local feature interaction and improved multi-scale feature representation, thereby enhancing the detection performance of defects with diverse textures, scales, and structural characteristics.

### 2.3 VP_CCFM module

In multi-scale object detection, features from different network layers differ in spatial resolution and semantic representation. Shallow features usually contain rich spatial details but relatively weak semantic information, whereas deep features provide stronger semantic representations with lower spatial resolution. Therefore, the main objective of feature fusion is to effectively reorganize hierarchical information by preserving discriminative features while suppressing redundant noise. Efficient feature interaction across different levels is important for improving discriminative representation, as the integration of global semantic information and local discriminative details can enhance feature robustness. Inspired by this idea, a VP_CCFM module is proposed at the feature fusion stage to improve the original CCFM in terms of both feature representation capability and computational efficiency.

From a feature-representation perspective, the VoVGSCSP module is introduced to replace the original RepC3 structure. RepC3 adopts a re-parameterization strategy, employing a multi-branch architecture during training to enhance feature representation, and merging these branches into a single convolutional operator during inference to improve computational efficiency (32). However, RepC3 mainly focuses on computational efficiency through structural re-parameterization and does not explicitly model cross-scale feature interactions. This may limit the effectiveness of feature aggregation when handling defect targets with diverse scales and complex appearance variations.

**Figure 5:**
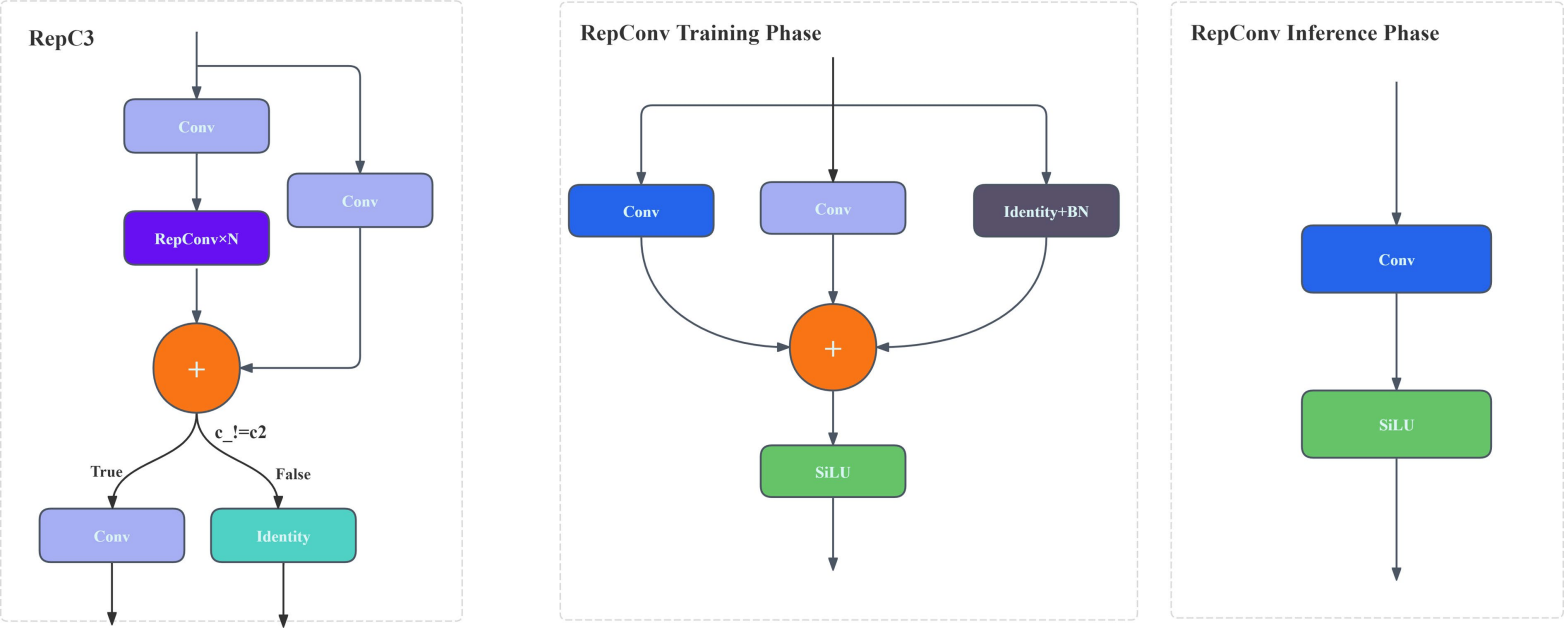
RepC3 module based on re-parameterization technology.

**Figure 6:**
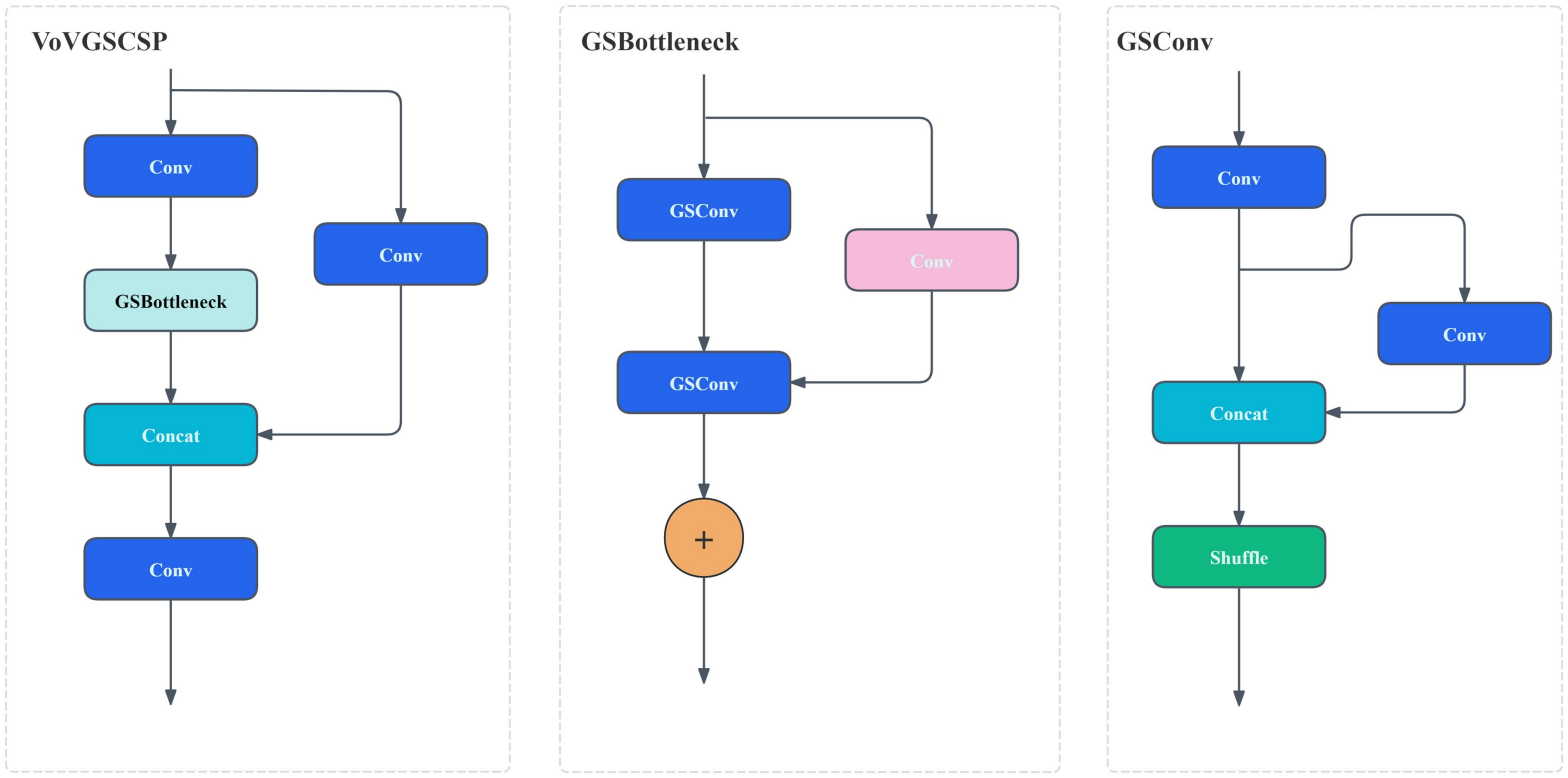
VoVGSCSP module based on GSConv.

In contrast, VoVGSCSP integrates the One-Shot Aggregation (OSA) mechanism from VoVNet with the Cross Stage Partial (CSP) architecture, while preserving the dual-branch structure during inference (33). Through feature splitting, the module separately strengthens local detail information and global semantic representations. OSA aggregates raw features across different layers via channel concatenation, thereby alleviating information compression and feature degradation (34). In addition, the introduced Group Shuffle Convolution (GSConv) combines standard convolution with depthwise separable convolution, reducing computational redundancy while maintaining effective channel interactions, thereby improving feature-representation efficiency. The structural differences between the RepC3 and VoVGSCSP modules are illustrated in Figures 5, 6.

**Figure 7:**
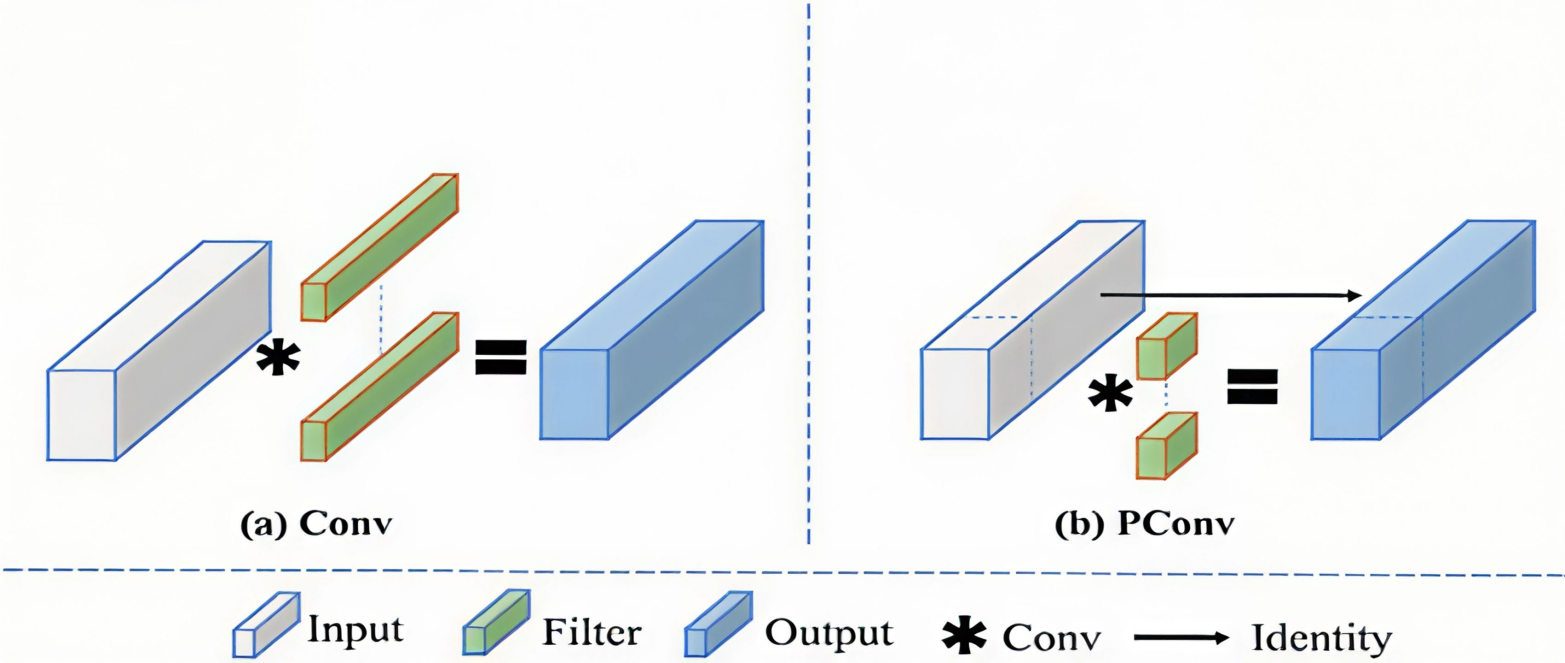
Structures of Conv and PConv.

To improve computational efficiency, Partial Convolution (PConv) is introduced as a replacement for standard convolution. PConv reduces the number of operations and memory usage. The core idea of PConv is to apply spatial convolution only to a subset of input channels, while the remaining channels are preserved through an identity mapping. The convolved and untouched feature channels are subsequently concatenated to form the output feature map (35). By exploiting inter-channel correlations, this strategy effectively reduces computational cost and memory access overhead while maintaining the capability to represent features. In this study, PConv is integrated into the VP_CCFM module to further improve the model’s computational efficiency, offering substantial savings in computation and memory. A comparison between the structures of standard convolution and PConv is presented in Figure 7.

Assuming the input and output feature maps of the standard convolution share the same spatial dimensions and channel numbers, its floating-point operations (FLOPs) and memory access cost (MAC) are given by Equations 2, 3.


$$F_{\text{Conv}}=h\times w\times k^{2}\times c^{2}$$ (2)

$$M_{\text{Conv}}=h\times w\times 2c+k^{2}\times c^{2}$$ (3)

The floating-point operations (FLOPs) and memory access cost (MAC) of Partial Convolution (PConv) are given by Equations 4, 5.


$$F_{\text{PConv}}=h\times w\times k^{2}\times c_{p}^{2}$$ (4)

$$M_{\text{PConv}}=h\times w\times 2c_{p}+k^{2}\times c_{p}^{2}$$ (5)

In these equations, h and w denote the height and width of the feature map, respectively; k denotes the kernel size; c denotes the total number of input and output channels; and c_p_ denotes the number of channels on which spatial convolution is performed. The remaining c-c_p_ channels are preserved through an identity mapping. Therefore, the output of the convolution branch contains c_p_ channels, and the concatenated output contains c channels in total.

According to Equations 2–5, when $c_{p}/c=1/2$ , PConv reduces the computational complexity and kernel-related memory access cost by approximately 75% compared with standard convolution, while the reduction in total memory access cost is dependent on the feature-map dimensions and channel configuration.

In summary, this study improves RT-DETR in three ways: frequency-domain feature decomposition, frequency-decoupled attention, and cross-scale feature fusion, thereby constructing a frequency–spatial collaborative modeling framework. Specifically, WT_Block enables a multi-scale frequency-domain representation, AIFI-HiLo enhances discriminative power for densely distributed small defects, and VP_CCFM improves feature fusion and information transmission efficiency. Through the joint contributions of these modules, the proposed framework enhances the detection performance of multi-scale surface defects on shawo radish while maintaining real-time inference.

## 3 Experiments and results analysis

### 3.1 Data acquisition

**Figure 8:**
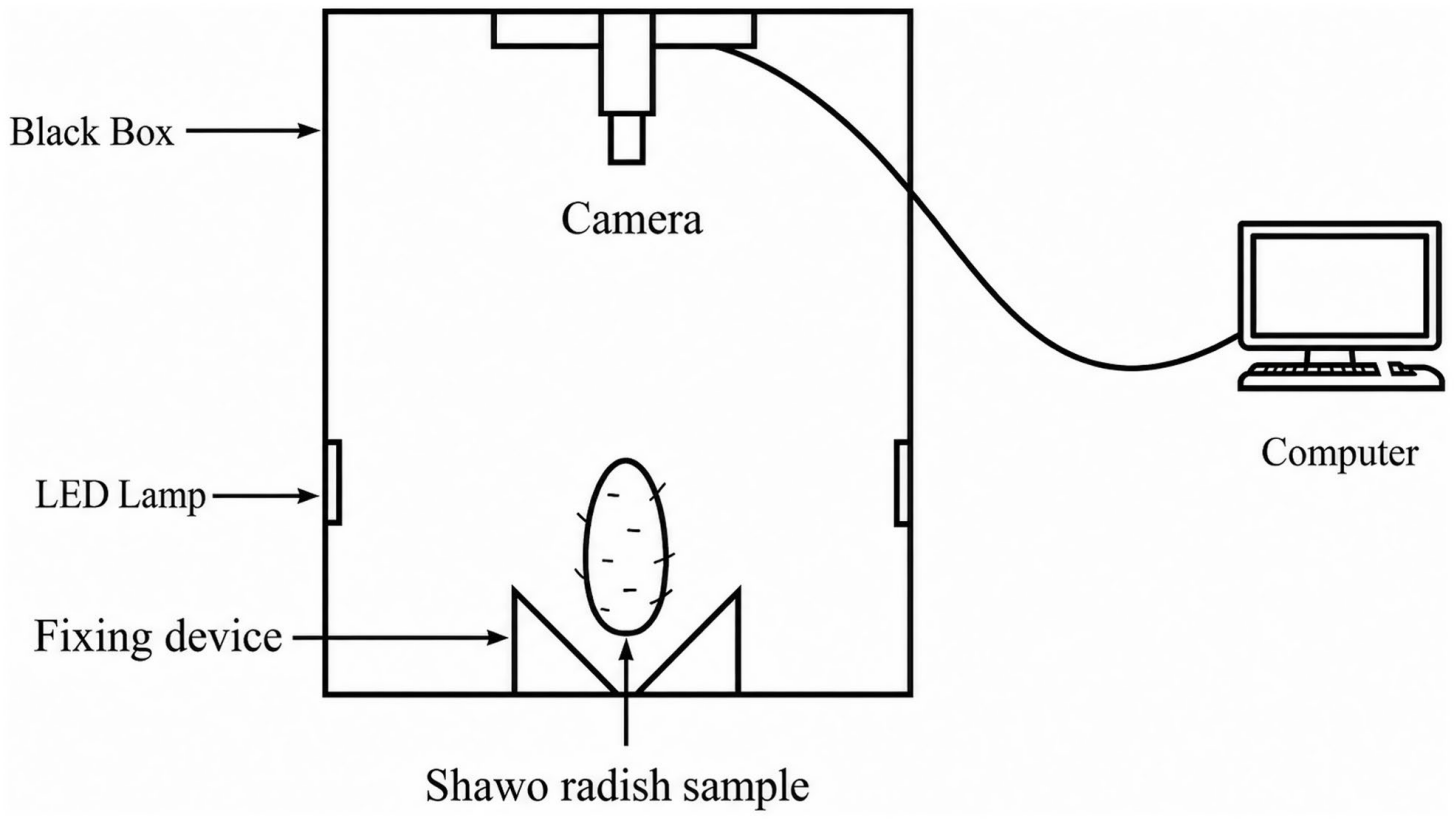
Image acquisition system.

Data were acquired from March to April 2025 at the standardized experimental field of the Tianjin Academy of Agricultural Sciences. The tested samples consisted of fresh shawo radish samples collected from a planting base in Xiqing District, Tianjin. The image acquisition system consisted of an industrial camera, a planar LED light source, and a fixed sample platform. Image acquisition was performed using a Daheng Imaging HN-3522-20 M-C1-1X industrial camera equipped with a 20-megapixel CMOS sensor and a 35 mm fixed-focus lens. An LED planar light source provided illumination at 5500 K. A schematic diagram of the image acquisition system is shown in Figure 8. The samples were manually rotated during acquisition to capture images from multiple viewing angles, ensuring comprehensive coverage of surface defect characteristics. The practical application scenario of this study is postharvest surface-defect inspection and quality grading of shawo radishes, which is generally performed in controlled or semi-controlled sorting environments. In such scenarios, extreme illumination changes, complex field backgrounds, and severe environmental disturbances are usually not present. Therefore, all images in this study were acquired under controlled illumination and a uniform background to approximate the intended application conditions and to reduce the influence of irrelevant environmental factors. This setting allowed a more focused evaluation of the proposed model improvements. After preprocessing and quality screening, 1,000 valid images were retained for model training, validation, and testing.

### 3.2 Dataset construction

**Figure 9:**
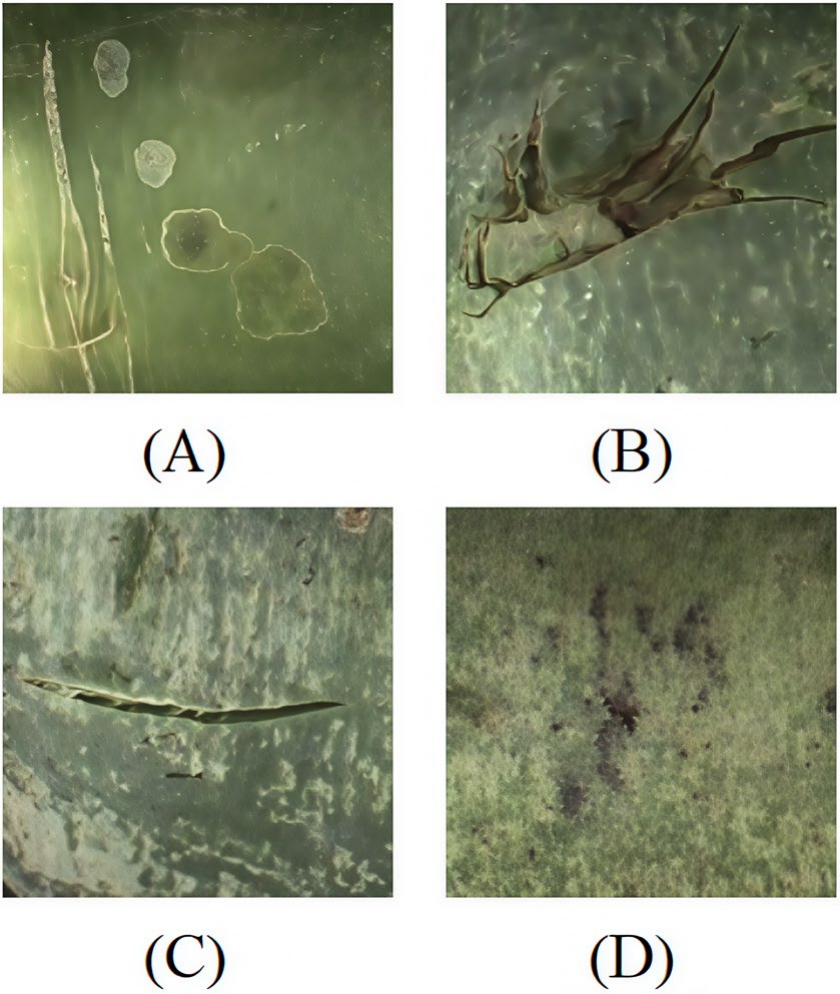
Examples of typical sample defects. **(A)** Growth nodule. **(B)** Mechanical damage. **(C)** Crack. **(D)** Disease spot.

To meet the quality control requirements for shawo radish grading and commercial distribution, surface defects were categorized into four classes according to practical market standards and defect characteristics: growth nodule (localized nodular protrusions associated with uneven physiological development), disease spot (dark lesions caused by diseases or environmental stress), mechanical damage (scratches or dents generated during harvesting or transportation), and crack (surface splitting or breakage of the epidermis). Based on the above classification criteria, all defect samples were annotated using the LabelMe tool in a standardized manner according to their defect categories. Representative defect samples are shown in Figure 9.

**Figure 10:**
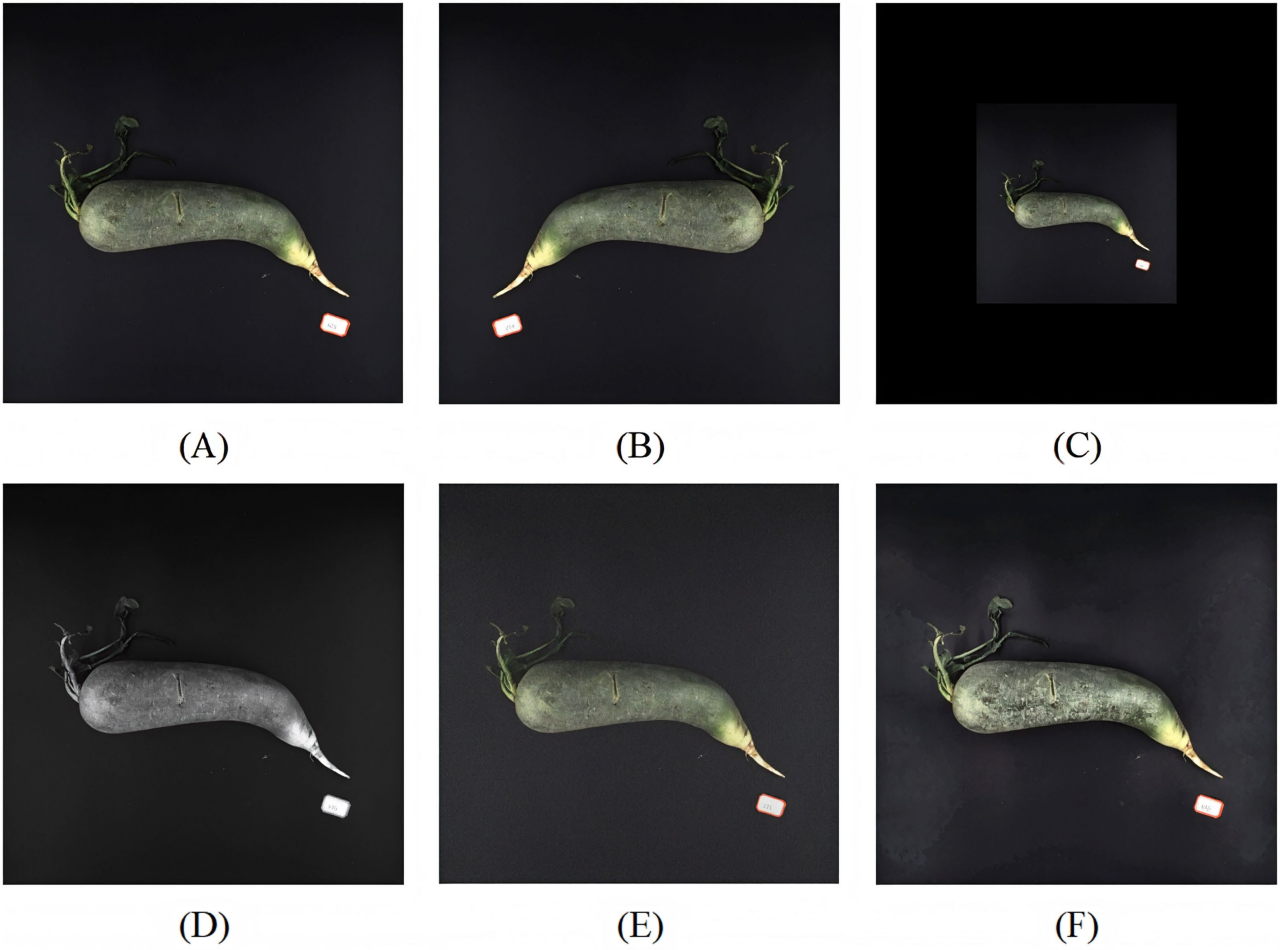
Effect of data augmentation. **(A)** Original image. **(B)** Flipping. **(C)** Scaling. **(D)** Color space conversion. **(E)** Noise addition. **(F)** Contrast enhancement.

To mitigate data leakage and ensure an objective evaluation, the original dataset of 1,000 images was split into training, validation, and test sets at a ratio of 6:2:2, yielding 600, 200, and 200 images, respectively. To further reduce the risk of sample-level data leakage, images captured from the same radish sample or the same acquisition source were assigned to the same subset rather than being split across the training, validation, and test sets. Subsequently, to enhance model generalization and robustness, a suite of data augmentation techniques—including image flipping, scaling, color space conversion, noise addition, and contrast enhancement—was applied exclusively to the training set. As a result, the training set was expanded to 1,200 images, while the validation and test sets remained unchanged at 200 images each. Examples of the augmented samples are shown in Figure 10.

To further clarify the sample sizes for each defect category and their distributions across the training, validation, and test subsets within the augmented dataset, comprehensive dataset statistics are presented in Table 1. The statistical results show that the defect categories maintain relatively balanced proportions within each data partition. This balanced partition ensures the fairness and stability of model training and evaluation, and further guarantees the credibility and reproducibility of subsequent experimental results.

**Table 1:** Distribution of annotated defect instances in the training, validation, and test sets.

Set	Growth nodule	Disease spot	Mechanical damage	Crack	Sum
Training set	1,170	1,143	1,206	1,008	4,527
Validation set	200	191	201	168	760
Test set	207	180	194	158	739

**Figure 11:**
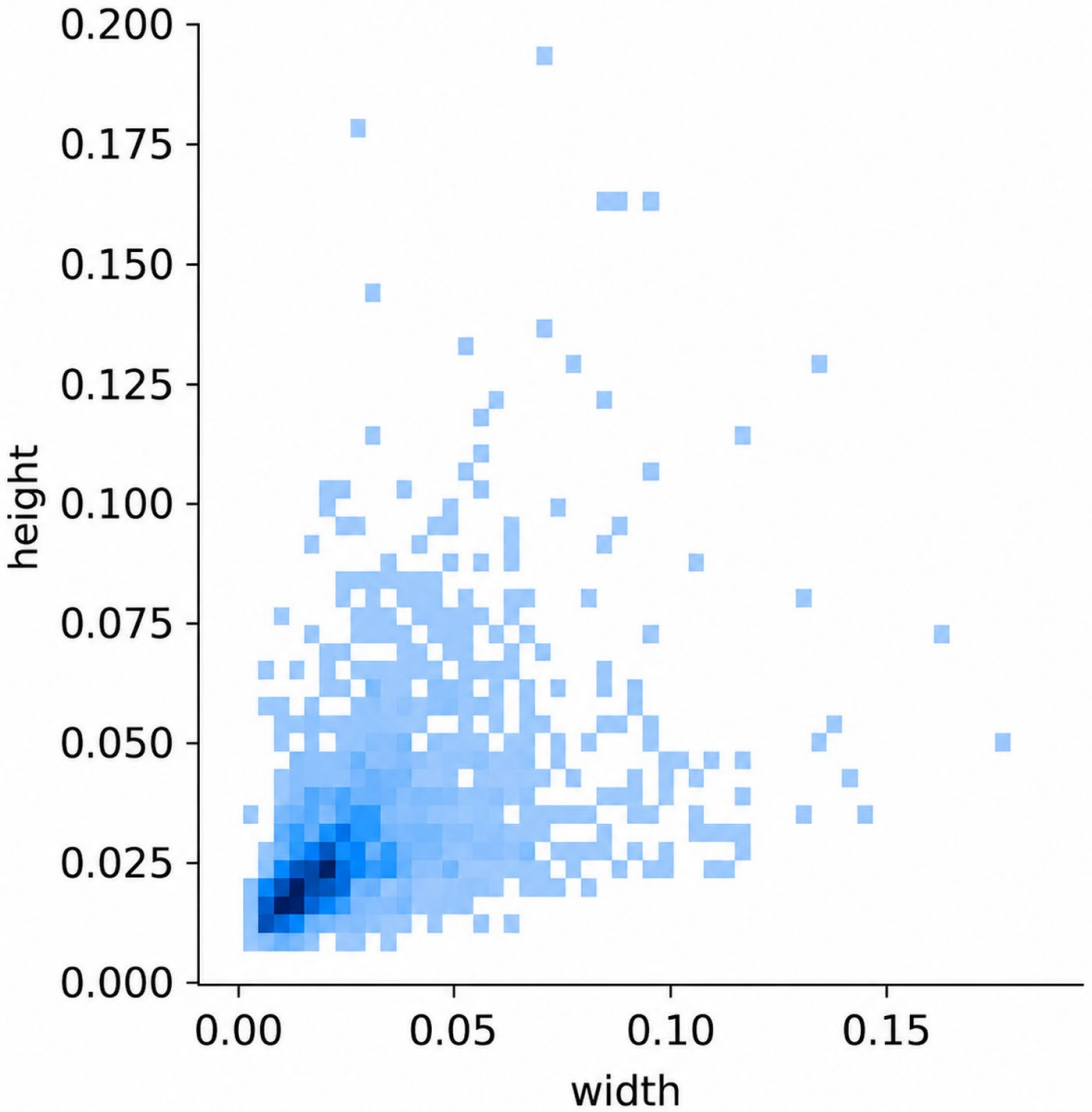
Size distribution of annotation boxes.

To further characterize the dataset, the size distribution of all annotated bounding boxes was statistically analyzed, as shown in Figure 11. The distribution is highly concentrated in the low-size region near the origin, indicating that small defect instances constitute the majority of the samples. Meanwhile, defects of varying sizes are also present, resulting in a pronounced multiscale distribution.

### 3.3 Experimental environment and parameter settings

During model training and testing, the experiments were conducted on a cloud computing platform equipped with an NVIDIA GeForce RTX 4090 GPU with 24 GB of video memory, 16 allocated virtual CPUs based on an Intel Xeon Platinum 8358P processor operating at 2.60 GHz, and 120 GB of system memory. The software environment consisted of Ubuntu 22.04, Python 3.10, PyTorch 2.1.2, and CUDA 11.8.

The main hyperparameters used during training were configured as follows: the input image size was uniformly resized to 640 × 640; the maximum number of training epochs was set to 600; the batch size was 8; the initial learning rate was 0.0001; and AdamW was used as the optimizer. During validation, the confidence threshold was set to 0.001, whereas during inference, it was set to 0.25. For performance evaluation, the Average Precision (AP) and mean Average Precision (mAP) metrics were calculated under an Intersection over Union (IoU) threshold of 0.5 (AP@50/mAP@50).

In addition, to mitigate the risk of overfitting under the relatively small self-constructed dataset and long training schedule, several regularization strategies were employed. Specifically, the weight decay coefficient was set to 0.0005, the dropout rate to 0.1, and an early stopping mechanism with a patience of 30 was adopted, meaning training was terminated if validation performance failed to improve for 30 consecutive epochs.

In the ablation experiments, all models were trained with identical hyperparameter configurations, including input resolution, optimizer, learning rate, batch size, training schedule and evaluation protocol, to construct strictly controlled experiments where only the modules proposed in this paper are taken as the sole variable. In the comparative experiments, all competing models were trained and evaluated using the same shawo radish surface defect dataset and the same training/validation/test split. The maximum number of training epochs was set to 600, and the input image size was set to 640 × 640 for all models. All models were initialized with their official pretrained weights when available and subsequently trained or fine-tuned on the training set used in this study. Other model-specific hyperparameters, such as optimizer settings, learning-rate schedules, and data preprocessing strategies, were configured according to their original implementations or commonly used settings in the literature to ensure stable convergence and fair performance comparison. All models were finally evaluated on the same test set using the same evaluation metrics, thereby improving the fairness and reproducibility of the experimental results.

### 3.4 Evaluation metrics

Precision, Recall, mean Average Precision at IoU threshold 0.5 (mAP@50), number of parameters (Parameters), computational complexity (Computation), and inference speed (FPS) were used as the evaluation metrics for shawo radish surface defect detection. The corresponding formulas for Precision, Recall, and mAP@50 are given in Equations 6–9.


$$\text{Precision}=\frac{\mathrm{TP}}{\mathrm{TP}+\mathrm{FP}}\times 100\%$$ (6)

$$\text{Recall}=\frac{\mathrm{TP}}{\mathrm{TP}+\mathrm{FN}}\times 100\%$$ (7)

$$\mathrm{AP}(k)=\int_{0}^{1}P_{k}(R)\mathrm{dR}\;\;(\mathrm{IoU}=0.5)$$ (8)

$$\mathrm{mAP}=\frac{1}{M}\sum_{k=1}^{M}\mathrm{AP}(k)$$ (9)

In these equations, TP denotes the number of positive samples correctly classified as positive, FN denotes the number of positive samples incorrectly classified as negative, and FP denotes the number of negative samples incorrectly classified as positive. AP(k) represents the average precision of the k -th defect category. At the same time, $P_{k}(R)$ denotes the proportion of true positive samples in the detection results at a Recall value of R . IoU=0.5 indicates that a prediction is considered a true positive only when the Intersection over Union (IoU) between the predicted bounding box and the ground-truth box exceeds 0.5. M represents the total number of defect categories to be detected.

### 3.5 Comparative experiments

To comprehensively assess the detection performance and computational efficiency of the proposed model, YOLOv5m, YOLOv8m, YOLOv11m, YOLOv26m, Faster R-CNN, DINO, Deformable DETR, and RT-DETR-r18 were selected as comparison models and evaluated on the same test set. The experimental results are summarized in Table 2. The evaluation metrics include Precision, Recall, mAP@50, parameter count, computational cost, and inference speed.

**Table 2:** Performance comparison of different detection models.

Model	Precision (%)	Recall (%)	mAP@50 (%)	Params (M)	GFLOPs	FPS
YOLOv5m	90.8	74.3	81.6	25.0	32.0	217
YOLOv8m	91.7	76.7	82.3	25.8	39.5	234
YOLOv11m	91.1	76.9	82.1	20.0	34.1	**242**
YOLOv26m	91.8	77.0	82.4	21.8	37.3	192
Faster R-CNN	81.9	78.0	80.4	41.3	175.0	45
DINO	88.0	76.9	82.6	46.8	118.9	56
Deformable DETR	89.2	77.1	82.7	40.0	170.0	57
RT-DETR-r18	91.6	78.4	83.4	20.1	29.1	145
OURS	**94.5**	**81.2**	**85.3**	**18.7**	**25.4**	105

Bold values indicate the best performance in the current column.

As shown in Table 2, the proposed model achieves the highest detection accuracy among all evaluated models, with Precision, Recall, and mAP@50 values of 94.5, 81.2, and 85.3%, respectively. Compared with the baseline RT-DETR-r18, the proposed model improves Precision, Recall, and mAP@50 by 2.9, 2.8, and 1.9 percentage points, respectively. These results suggest that WT_Block, AIFI-HiLo, and VP_CCFM effectively enhance feature representation for shawo radish surface defect detection. The proposed model also outperforms the YOLO series in detection accuracy, as YOLOv5m, YOLOv8m, YOLOv11m, and YOLOv26m all show lower mAP@50 values. Although YOLO-based models demonstrate strong real-time inference capability, their feature representation remains limited when dealing with small-scale, weakly textured, and highly similar defect categories.

Compared with two-stage detectors and Transformer-based detectors, the proposed model also demonstrates clear advantages. Faster R-CNN achieves a Precision of 81.9% and an mAP@50 of 80.4%, indicating that it is more susceptible to interference from complex surface textures and more prone to false positive predictions. DINO and Deformable DETR achieve mAP@50 values of 82.6 and 82.7%, respectively, but both remain lower than that of the proposed model. These findings indicate that relying solely on global attention or deformable attention is insufficient to address weak textures, slender cracks, and multi-scale defect patterns in shawo radish surface defect detection. By contrast, the proposed frequency–spatial collaborative modeling strategy enhances the discriminative power of defect features and improves overall detection performance.

Regarding model complexity, the proposed model contains 18.7 million parameters and requires 25.4 GFLOPs, representing reductions of approximately 7.0 and 12.7%, respectively, compared with the baseline RT-DETR-r18, which has 20.1 million parameters and 29.1 GFLOPs. This demonstrates that lightweight structures such as VP_CCFM can reduce redundant computation while maintaining detection accuracy. In addition, compared with Faster R-CNN, DINO, and Deformable DETR, the proposed model shows lower parameter count and computational cost, reflecting better lightweight characteristics.

In terms of inference speed, YOLO-based models achieve higher FPS values, with YOLOv11m, YOLOv8m, and YOLOv5m reaching 242, 234, and 217 FPS, respectively. The proposed model achieves 105 FPS, which is lower than that of the YOLO series but substantially higher than those of Faster R-CNN, DINO, and Deformable DETR, which achieve 45, 56, and 57 FPS, respectively. These results indicate that the proposed model maintains favorable real-time inference capability while achieving superior detection accuracy, thus providing a balanced trade-off among accuracy, model complexity, and inference efficiency.

**Figure 12:**
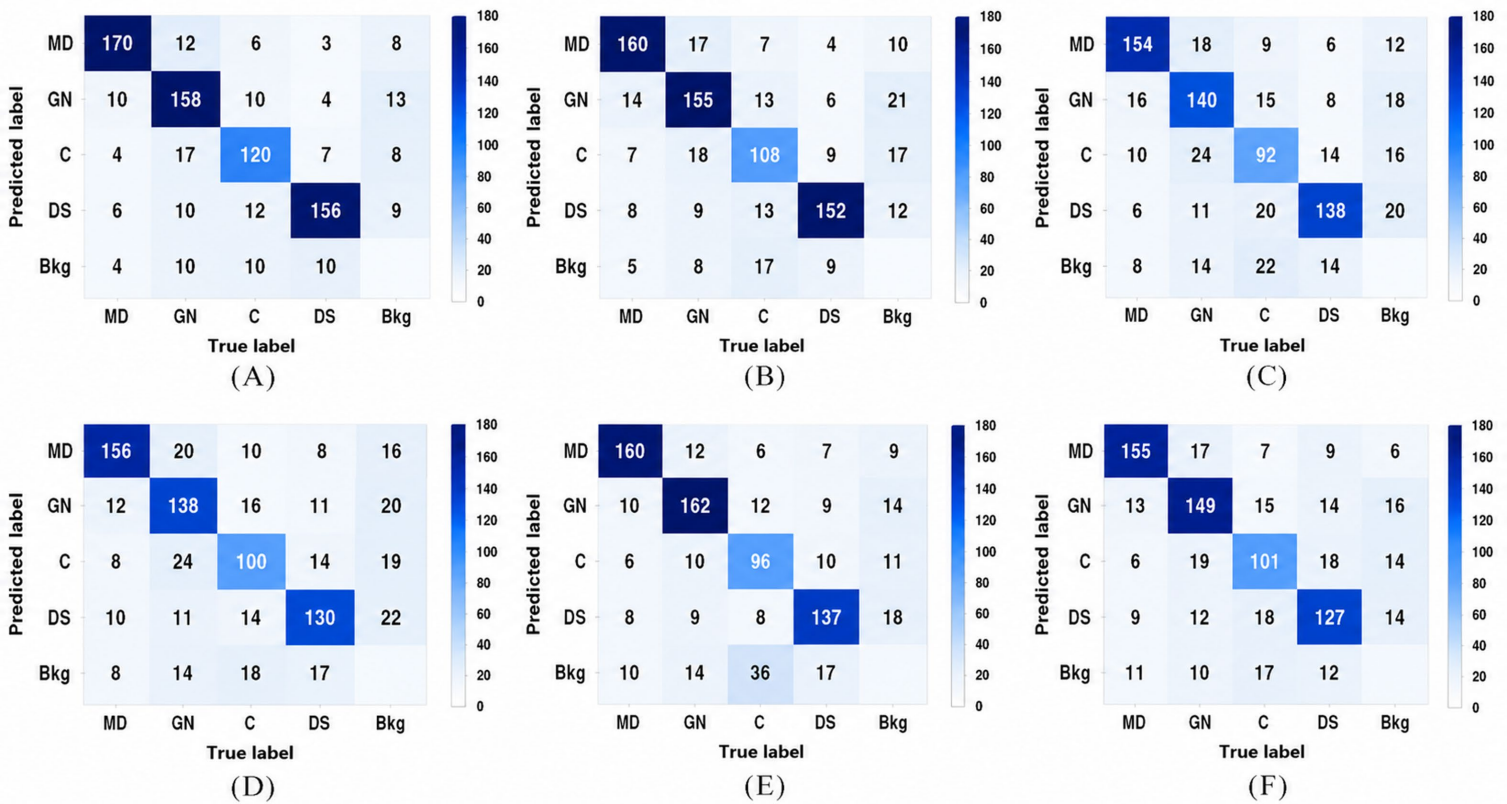
Confusion matrices of different detection models for Shawo radish surface defect detection: **(A)** OURS. **(B)** RT-DETR-r18. **(C)** YOLOv26m. **(D)** Faster R-CNN. **(E)** DINO. **(F)** Deformable DETR. MD, GN, C, DS, and Bkg represent Mechanical Damage, Growth Nodule, Crack, Disease Spot, and Background, respectively.

To further analyze false positives and missed detections, confusion matrices were generated for the proposed model, RT-DETR-r18, YOLOv26m, Faster R-CNN, DINO, and Deformable DETR, as shown in Figures 12A–F. In each confusion matrix, the horizontal axis denotes the true labels, while the vertical axis denotes the predicted labels. The Background row represents missed detection samples, whereas the Background column represents false positive samples.

The confusion matrix of the proposed model shows the most concentrated diagonal distribution, indicating more stable recognition across the four defect categories. It correctly identifies 170 mechanical damage, 158 growth nodule, 120 crack, and 156 disease spot instances, exceeding the corresponding results of RT-DETR-r18 (160, 155, 108, and 152). The improvement is especially notable for crack detection, where correct identifications increase from 108 to 120, outperforming YOLOv26m, Faster R-CNN, DINO, and Deformable DETR.

The proposed model produces 34 missed detections in total, fewer than RT-DETR-r18, YOLOv26m, Faster R-CNN, DINO, and Deformable DETR, which yield 39, 58, 57, 77, and 50, respectively. Although RT-DETR-r18 has slightly fewer missed growth nodule and disease spot instances, the proposed model achieves the lowest overall missed-detection count and misses only 10 crack instances. For false positives, the proposed model generates 38 background false positives, lower than the totals of all comparison models. The reduction is particularly evident for growth nodule and crack, indicating improved discrimination between true defects and visually similar surface textures. Overall, the proposed model achieves the best balance among correct recognition, missed-detection reduction, and false-positive suppression.

**Figure 13:**
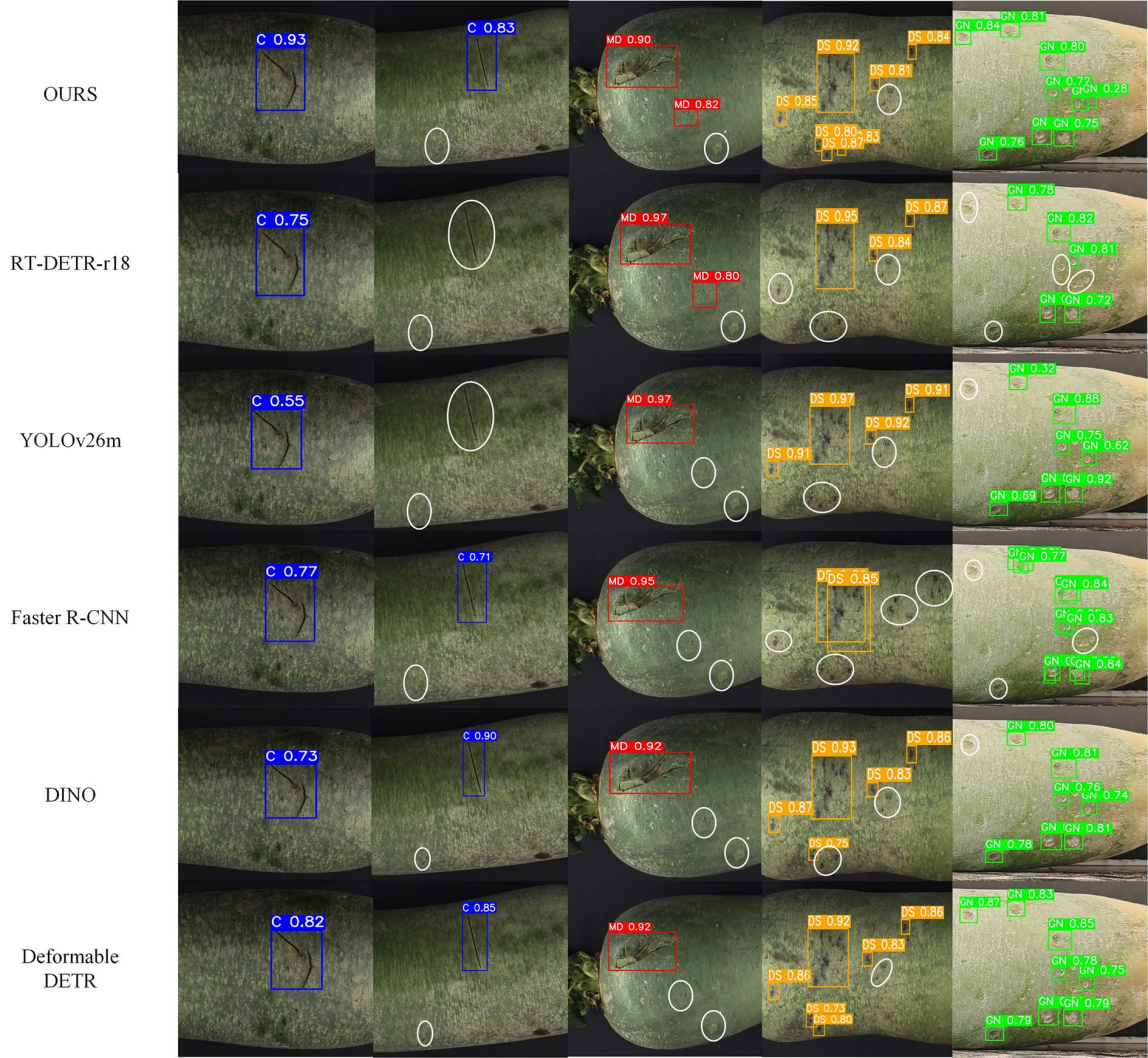
Visualization comparison of surface defect detection results of shawo radishes using different models. MD, Mechanical Damage; GN, Growth Nodule; C, Crack; DS, Disease Spot.

To more intuitively demonstrate the practical detection performance of different models, representative test samples were selected for visual comparison, as shown in Figure 13. Each row corresponds to one detection model, and each column represents a typical sample of a specific defect category. Colored bounding boxes indicate successfully detected defect targets, while the category abbreviation and confidence score above each box represent the predicted category and confidence value. White ellipses mark ground-truth defect regions that the corresponding model fails to detect, namely missed targets.

As shown in Figure 13, the proposed model achieves favorable detection results across different defect types, with more accurate localization and higher confidence scores. For crack defects, the proposed model effectively captures relatively obvious slender crack regions, whereas several comparison models produce low-confidence predictions or missed detections. It should be noted that a small number of tiny cracks with weak edge responses and low contrast against the radish surface texture are still difficult to detect, even for the proposed model. For mechanical damage and disease spot, all models can roughly detect the main defective regions, but the proposed model performs better in terms of localization completeness and confidence stability. For growth nodule defects, which are usually small, dense, and unevenly distributed, comparison models often suffer from missed detections or incomplete bounding boxes, whereas the proposed model can identify more small nodules and demonstrates stronger capability for dense small-object detection.

From the visual detection results, white ellipse regions can be observed in the outputs of RT-DETR-r18, YOLOv26m, Faster R-CNN, DINO, and Deformable DETR, indicating that these models still miss some weakly textured, small-scale, or low-contrast defects. In comparison, the proposed model produces fewer missed regions, demonstrating stronger Recall capability under challenging defect appearance conditions. This observation is consistent with the quantitative results in Table 2 and the confusion matrix analysis in Figure 12, further verifying the effectiveness of WT_Block, AIFI-HiLo, and VP_CCFM in frequency-domain feature enhancement, high–low frequency attention modeling, and cross-scale feature fusion.

In summary, the proposed model demonstrates strong overall performance in quantitative evaluation, confusion matrix analysis, and visual detection comparison. Compared with the comparison models, the proposed model improves Precision, Recall, and mAP@50 while reducing parameter count and computational cost. It also alleviates false positives and missed detections in complex defect scenarios. These results indicate that the constructed frequency–spatial collaborative modeling framework effectively improves the accuracy, robustness, and real-time performance of shawo radish surface defect detection.

### 3.6 Ablation experiments

A rigorous ablation study was conducted to decouple and evaluate the individual contribution of each integrated module. Following a strictly controlled-variable strategy, the baseline RT-DETR-r18 model was incrementally modified while keeping the dataset partitioning, hyperparameter configurations, and training settings unchanged. To reduce the influence of random variation and improve the reliability of the conclusions, all models were trained and evaluated using three predefined random seeds, namely 42, 2023, and 2024. The corresponding performance metrics are presented in Table 3, where the values represent the mean results obtained from three runs, with the standard deviations shown as subscripts.

**Table 3:** Quantitative metric analysis of the ablation study.

Exp.	WT_Block	VP_CCFM	AIFI-HiLo	Precision (%)	Recall (%)	mAP@50 (%)	Params (M)	GFLOPs	FPS
1	-	-	-	91.6_0.2_	78.4_0.3_	83.4_0.2_	20.1	29.1	**145**_**3.3**_
2	√	-	-	92.1_0.3_	79.2_0.4_	84.5_0.3_	18.9	28.0	134_2.0_
3	-	√	-	92.5_0.3_	79.0_0.4_	84.0_0.3_	19.0	26.8	117_1.8_
4	-	-	√	93.0_0.4_	79.5_0.4_	84.6_0.3_	20.0	29.1	110_4.5_
5	√	√	-	94.1_0.3_	80.4_0.5_	85.0_0.4_	**18.5**	26.5	111_2.9_
6	√	-	√	93.9_0.2_	**82.3**_0.3_	84.3_0.4_	19.7	30.4	98_3.5_
7	-	√	√	91.3_0.4_	80.4_0.4_	84.1_0.2_	19.1	26.6	120_2.7_
8	√	√	√	**94.5**_0.4_	81.2_0.5_	**85.3**_0.4_	18.7	**25.4**	105_3.0_

The values indicate the mean results obtained from three random seeds, with the standard deviations shown as subscripts. Bold values indicate the best performance in the current column.

Compared with the baseline, WT_Block alone improves Precision, Recall, and mAP@50 by 0.5, 0.8, and 1.1 percentage points, respectively, indicating that frequency-domain decomposition contributes to enhanced local texture and structural representation. Introducing VP_CCFM independently increases Precision, Recall, and mAP@50 by 0.9, 0.6, and 0.6 percentage points, respectively, while reducing both parameters and GFLOPs, demonstrating its effectiveness in lightweight multi-scale feature fusion. AIFI-HiLo alone yields improvements of 1.4, 1.1, and 1.2 percentage points in Precision, Recall, and mAP@50, respectively, suggesting that high–low frequency attention strengthens defect-related feature selection.

The two-module combinations further demonstrate the complementary roles of these modules. WT_Block + VP_CCFM achieves 94.1% Precision, 80.4% Recall, and 85.0% mAP@50, outperforming either module used alone and indicating that frequency-domain enhancement and efficient multi-scale fusion can provide synergistic benefits. WT_Block + AIFI-HiLo obtains the highest Recall of 82.3%, suggesting improved target coverage and reduced missed detections; however, its mAP@50 remains lower than that of the full model, implying that additional cross-scale fusion is still necessary for balanced performance. VP_CCFM + AIFI-HiLo improves Recall and mAP@50 over the baseline but shows a slight decrease in Precision, suggesting that the absence of WT_Block may weaken fine-grained representation for subtle defect regions.

When all three modules are integrated, the proposed model achieves the best overall performance, with Precision, Recall, and mAP@50 reaching 94.5, 81.2, and 85.3%, respectively. Compared with the baseline, these results correspond to improvements of 2.9, 2.8, and 1.9 percentage points, while parameters and GFLOPs are reduced by 7.0 and 12.7%, respectively. These findings confirm that WT_Block, AIFI-HiLo, and VP_CCFM provide complementary contributions in frequency-domain representation, attention-based feature selection, and cross-scale feature fusion, enabling a better balance between detection accuracy and computational efficiency. It should be noted that the ablation results mainly demonstrate performance-level complementarity, while feature-level interactions are further examined through visualization experiments.

**Figure 14:**
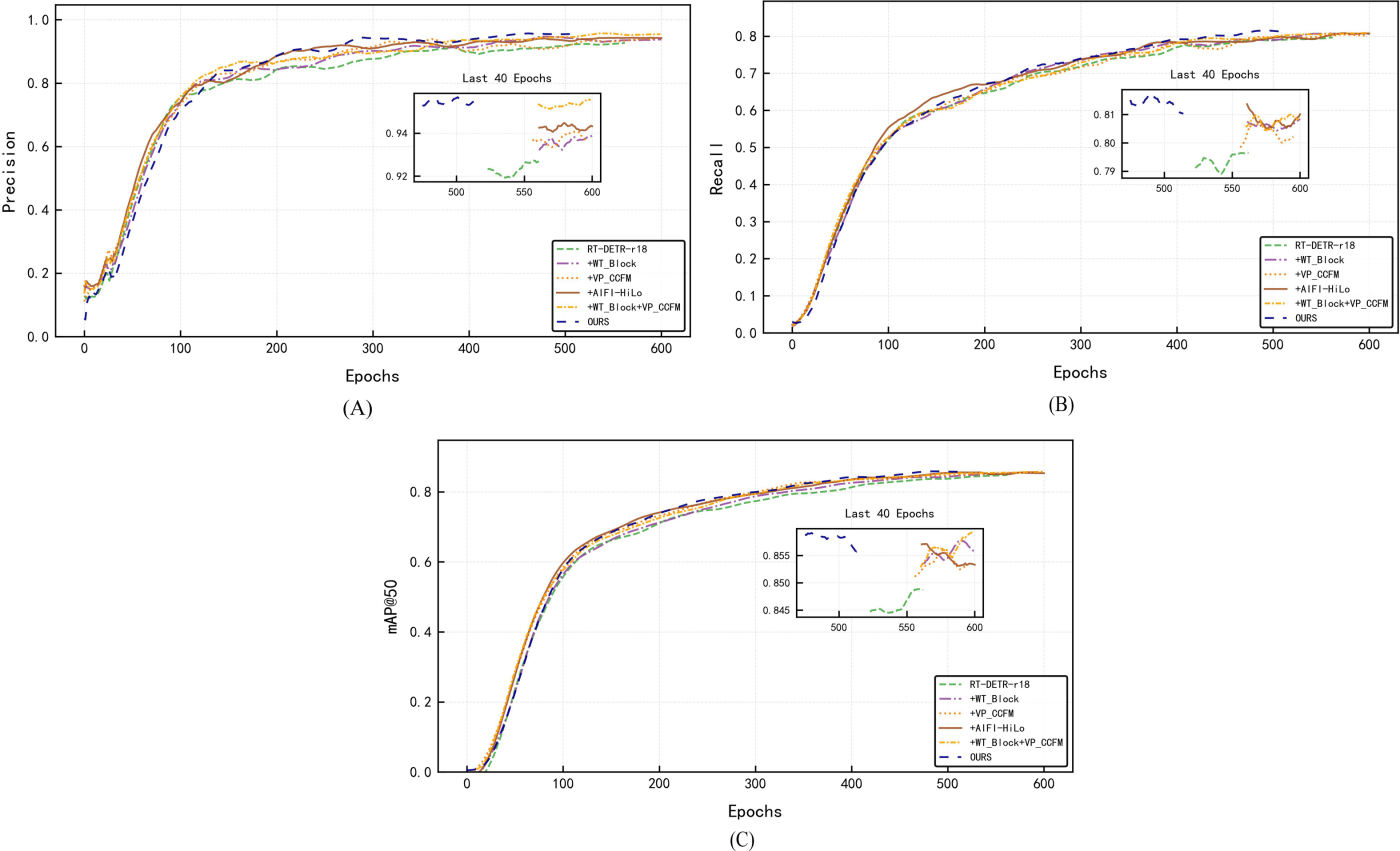
Training convergence curves of ablation models. **(A)** Precision. **(B)** Recall. **(C)** mAP@50.

To further examine the training dynamics of the ablation models, the convergence curves of Precision, Recall, and mAP@50 obtained under the random seed of 42 were selected for representative analysis, as shown in Figures 14A–C. Overall, all models show rapid performance improvement during the early training stage, followed by gradual stabilization in the later epochs, indicating that the models can converge normally under the same training settings. As shown in Figure 14A, the proposed model maintains higher Precision than the other ablation variants during most training epochs and achieves the best final Precision, suggesting stronger classification capability and more stable optimization. For Recall in Figure 14B, the models exhibit a similar convergence trend. The variant with AIFI-HiLo shows relatively high Recall during training, indicating that high–low frequency attention may help improve target coverage and reduce missed detections. Nevertheless, the proposed model provides the most favorable overall balance among Precision, Recall, and mAP@50, indicating superior comprehensive detection performance. Figure 14C shows the convergence process of mAP@50. Although the differences among some variants are relatively small, the proposed model consistently maintains superior mAP@50 in the later training stage and ultimately achieves the best overall detection accuracy. The enlarged views of the last 40 epochs further confirm that the proposed model achieves superior Precision and mAP@50 while maintaining a competitive Recall, demonstrating the best overall balance among the evaluated metrics.

**Figure 15:**
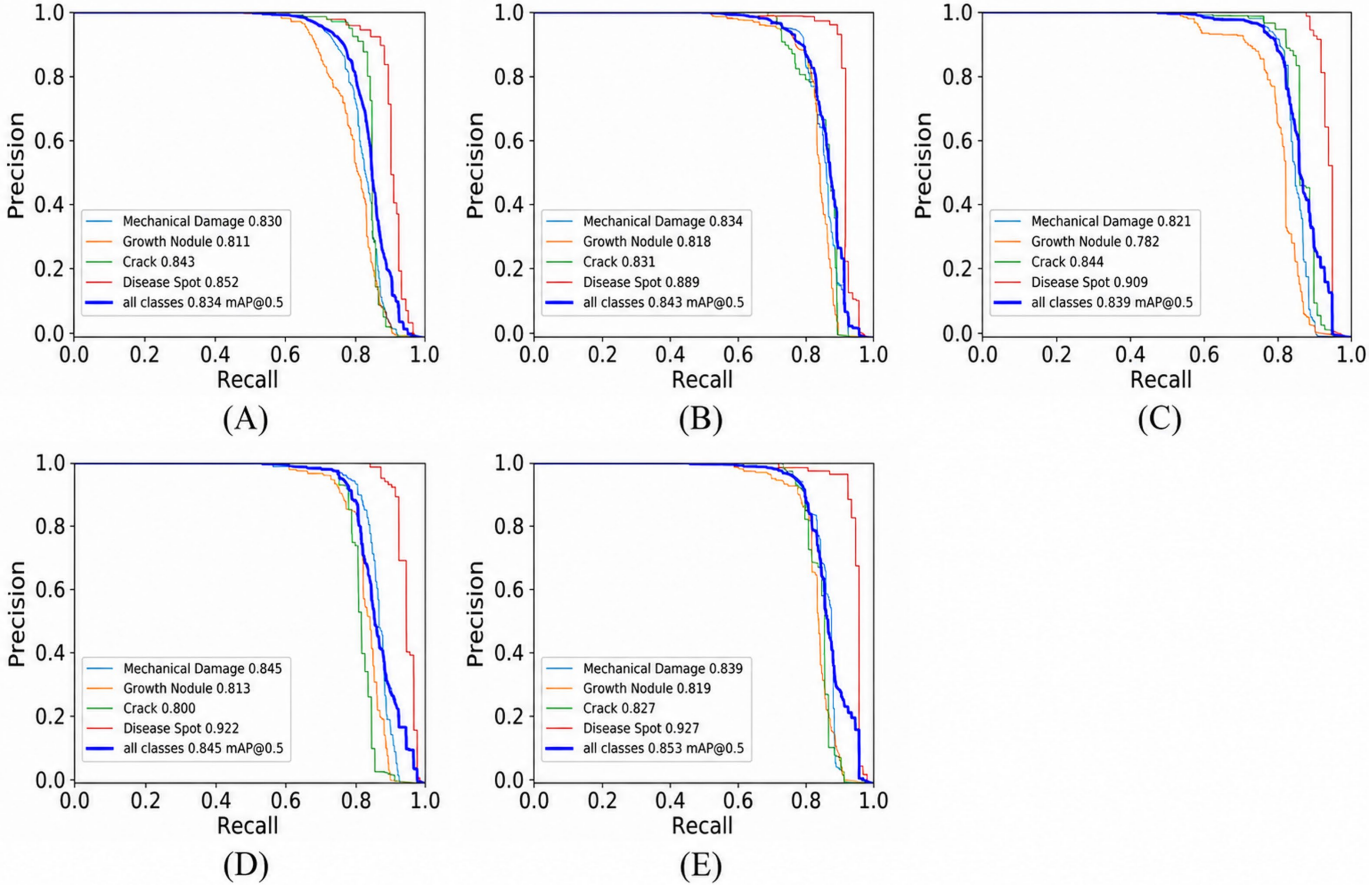
Precision–recall curves for four defect categories under selected ablation models. **(A)** RT-DETR-r18. **(B)** + WT_Block. **(C)** + VP_CCFM. **(D)** + AIFI-HiLo. **(E)** Ours.

To provide a more intuitive and quantitative assessment of the contribution of each proposed module to different defect categories, precision–recall curves obtained under the random seed of 42 are presented in Figures 15A–E. Overall, the proposed modules improve category-level performance to varying degrees, and their effects vary with the texture complexity, structural characteristics, and scale distribution of different defects. As shown in Figure 15A, the baseline RT-DETR-r18 achieves an overall mAP@50 of 0.834. Among the four defect categories, disease spot obtains a relatively high AP of 0.852 due to its more obvious color and texture characteristics, whereas growth nodule shows the lowest AP of 0.811, indicating that its irregular morphology, dense distribution, and scale variation make accurate detection more difficult. After introducing WT_Block, the overall mAP@50 increases to 0.843, as shown in Figure 15B. AP improvements are observed for mechanical damage, growth nodule, and disease spot, with disease spot showing the largest improvement of 3.7 percentage points. This result is consistent with the design of WT_Block, suggesting that wavelet-based frequency-domain decomposition helps enhance structural and texture representations of defect regions. As shown in Figure 15C, VP_CCFM improves the overall mAP@50 to 0.839. Notably, crack achieves an AP of 0.844, the best result for this category among the single-module variants. This may be attributed to the enhanced cross-scale feature interaction and aggregation capability of VP_CCFM, which helps preserve contour and scale-related information. However, the AP for growth nodule remains relatively low, indicating that feature fusion alone may be insufficient for defects with weak local textures and complex morphology. As shown in Figure 15D, AIFI-HiLo achieves the highest single-module mAP@50 of 0.845. The AP for disease spot increases to 0.922, representing a 7.0 percentage-point improvement over the baseline. This indicates that high–low frequency attention helps balance local detail modeling and global contextual representation, thereby improving feature discriminability for defects with complex textures. However, the AP for crack decreases slightly, suggesting that attention modeling alone may not fully preserve continuous edge structures. As shown in Figure 15E, the proposed model achieves the highest overall mAP@50 of 0.853 and shows relatively balanced performance across defect categories. The AP values for mechanical damage, growth nodule, crack, and disease spot are 0.839, 0.819, 0.827, and 0.927, respectively. Although the AP for crack is slightly lower than that of the baseline and the VP_CCFM variant, the proposed model achieves the best overall performance, indicating that WT_Block, AIFI-HiLo, and VP_CCFM provide complementary benefits in frequency-domain representation, global–local feature modeling, and cross-scale feature fusion.

To further examine the impact of each improved module on different defect categories, a grouped dot plot of per-category AP values was constructed, as shown in Supplementary Figure 1. The results show that the contribution of each module varies across defect categories, reflecting their different roles in feature representation, attention modeling, and multi-scale fusion. For mechanical damage, the AIFI-HiLo variant achieves the highest AP of 84.5%, which is 1.5 percentage points higher than the baseline. The proposed model also improves the AP to 83.9%, suggesting that frequency-aware attention helps capture local structural variations and edge-related features. For growth nodule, the proposed model obtains the best AP of 81.9%, slightly higher than AIFI-HiLo and the baseline. Since growth nodule defects usually exhibit irregular morphology, scale variation, and weak local texture, this improvement indicates that the combination of frequency-domain representation, attention modeling, and feature fusion is beneficial for complex defect representation. For crack, the VP_CCFM variant achieves the highest AP of 84.4%, slightly exceeding the baseline value of 84.3%, whereas the proposed model obtains an AP of 82.7%. This may be because crack defects are slender and continuous, making their detection highly dependent on cross-scale information propagation and edge-contour preservation. Although the AP of the proposed model decreases slightly for this category, its overall mAP@50 remains the highest among all configurations, indicating that the local decline in a single category does not undermine the overall performance improvement. For disease spot, the proposed model achieves the highest AP of 92.7%, which is 7.5 percentage points higher than the baseline and represents the most significant improvement among all defect categories. This result suggests that frequency-domain information modeling and high–low frequency attention are effective in enhancing discriminative texture and structural features for disease-spot detection.

To further analyze the feature-level effects of the proposed modules, Grad-CAM visualizations were performed on representative defect samples, as shown in Supplementary Figure 2, where columns A–D correspond to growth nodule, disease spot, crack, and mechanical damage, respectively. Compared with the baseline RT-DETR-r18, the single-module variants exhibit different activation characteristics. WT_Block enhances responses around local texture discontinuities and defect boundaries, especially for growth nodule and crack, suggesting that frequency-domain decomposition may help capture fine-grained structural variations. VP_CCFM produces broader activation regions, which is consistent with its role in multi-scale feature fusion, although some background regions are also activated. In contrast, AIFI-HiLo generates more concentrated responses around discriminative defect areas, particularly in crack and growth nodule samples, indicating its advantage in feature selection and irrelevant-response suppression.

When the three modules are integrated, the proposed model produces more complete and defect-relevant activation patterns in most cases. For growth nodule and crack, the high-response regions are more consistent with the annotated defect areas, indicating improved sensitivity to small protrusions, boundary changes, and slender structural defects. For disease spot and mechanical damage, the proposed model maintains stronger defect-related responses while reducing irrelevant background activation compared with some single-module variants. These results suggest that WT_Block, AIFI-HiLo, and VP_CCFM play complementary roles in frequency-domain representation, attention-guided feature selection, and multi-scale feature fusion. Combined with the quantitative ablation results, the Grad-CAM analysis further supports the effectiveness of the proposed module combination for shawo radish surface defect detection.

Overall, the quantitative and visual results jointly verify the effectiveness of the proposed module combination. WT_Block contributes to frequency-domain feature enhancement, AIFI-HiLo strengthens discriminative feature selection through high–low frequency attention, and VP_CCFM improves lightweight multi-scale feature fusion. The complementary effects among these modules enable the proposed model to achieve the best overall mAP@50 while maintaining lower parameters and GFLOPs than the baseline. Meanwhile, the convergence curves, per-category PR curves, AP distributions, and Grad-CAM visualizations further confirm that the proposed model improves both performance-level metrics and feature-level defect responses. These findings demonstrate that the proposed architecture is effective for detecting shawo radish surface defects with complex morphology, weak texture, and scale variation.

### 3.7 Robustness under simulated perturbations

To further assess the generalization robustness of the proposed model under possible interference conditions in practical sorting scenarios, simulated disturbance test sets were constructed using data augmentation. Three typical disturbances were considered, including uneven illumination, dust-like occlusion, and motion blur, each of which was designed at light and heavy levels by adjusting the perturbation intensity. Accordingly, six disturbed test sets were constructed: light uneven illumination, heavy uneven illumination, light dust-like occlusion, heavy dust-like occlusion, light motion blur, and heavy motion blur. The simulation was performed on the 200 images in the original test set. As the applied disturbances only modify image appearance and do not change image size or object locations, the original labels were directly reused. Representative disturbed samples are presented in Supplementary Figure 3. To ensure the repeatability and consistency of the simulated disturbance test sets, the perturbation parameters for the light and heavy disturbance levels were predefined, as summarized in Table 4.

**Table 4:** Parameter settings for light and heavy simulated disturbance levels.

Disturbance type	Parameter	Light disturbance	Heavy disturbance
Dust-like occlusion	Coverage ratio	0.005–0.015	0.025–0.060
	Dust spot radius	2–6 px	4–14 px
	Blending alpha	0.30–0.50	0.50–0.75
	Edge-smoothing kernel	3	5
Uneven illumination	Brightness range	0.80–1.20	0.55–1.45
	Spot strength	0.15–0.25	0.30–0.45
	Sigma range	0.25–0.40	0.25–0.50
Motion blur	Kernel size range	8–13	17–23

Coverage ratio represents the proportion of the image area covered by dust-like occlusion. Dust spot radius controls the size of dust or stain regions. Blending alpha denotes the fusion weight between the disturbance layer and the original image. Edge-smoothing kernel is used to soften dust boundaries. Brightness range controls global illumination variation, spot strength determines the intensity of local bright or dark regions, and sigma range controls the spatial spread of illumination spots. Kernel size range determines the strength of motion blur.

As shown in Supplementary Figure 4, different simulated disturbances cause different degrees of mAP@50 degradation for both models. Under dust-like occlusion, light disturbance leads to moderate mAP@50 decreases of 2.7 and 3.2 percentage points for RT-DETR-r18 and the proposed model, respectively. However, under heavy dust-like occlusion, the mAP@50 values drop sharply by 35.0 and 35.3 percentage points. This indicates that severe dust-like occlusion has the strongest impact on detection performance, mainly because dense occlusion can directly cover defect regions and destroy discriminative visual cues, such as local texture, color contrast, and defect boundaries. In contrast, uneven illumination has a relatively limited influence on both models. Under light uneven illumination, the mAP@50 values decrease by only 0.5 and 0.9 percentage points, respectively, while under heavy uneven illumination, the decreases are 2.9 and 2.4 percentage points. This may be because illumination variation mainly changes the global brightness distribution but does not directly damage the spatial structure or boundary continuity of defect regions. Therefore, effective defect-related structural and texture features can still be extracted. For motion blur, light disturbance causes only slight performance degradation, with mAP@50 reductions of 0.4 and 0.8 percentage points for RT-DETR-r18 and the proposed model, respectively. Under heavy motion blur, the decreases become more obvious, reaching 8.4 and 7.3 percentage points. This is because motion blur weakens edge sharpness and high-frequency texture responses, which are important for defect localization and classification. Compared with dust-like occlusion, motion blur does not completely remove defect information, but it reduces the clarity of defect boundaries. Overall, dust-like occlusion causes the most significant degradation, followed by motion blur, whereas uneven illumination has the smallest impact. Although the proposed model does not show lower attenuation than the baseline under all light disturbance conditions, it consistently maintains higher absolute mAP@50 values across all simulated scenarios. In particular, under heavy uneven illumination and heavy motion blur, the proposed model shows smaller attenuation than RT-DETR-r18, indicating better robustness when defect details are degraded but not completely occluded.

Table 5 summarizes the mAP@50 attenuation of RT-DETR-r18 and the proposed model under different simulated disturbance conditions. Light uneven illumination and light motion blur cause only minor performance degradation for both models, indicating that mild brightness variation and slight blur have limited influence on detection performance. In contrast, heavy dust-like occlusion leads to the most severe attenuation, with mAP@50 decreases of 35.0 and 35.3 percentage points for RT-DETR-r18 and OURS, respectively. This is mainly because dense occlusion directly covers defect regions and weakens key visual cues such as texture, contrast, and boundaries.

**Table 5:** Attenuation of mAP@50 under different disturbance conditions.

Disturbance type	RT-DETR-r18 (PP)	OURS (PP)
Clean	0.0	0.0
Light dust-like occlusion	2.7	3.2
Heavy dust-like occlusion	35.0	35.3
Light uneven illumination	0.5	0.9
Heavy uneven illumination	2.9	2.4
Light motion blur	0.4	0.8
Heavy motion blur	8.4	7.3

mAP@50 attenuation was calculated as mAP@50(clean) − mAP@50(disturbed). The attenuation values for the clean test set were defined as 0 for both models. A larger attenuation value indicates a greater performance decrease. For example, under light dust-like occlusion, the attenuation values of RT-DETR-r18 and OURS were 83.4–80.7 = 2.7 PP and 85.3–82.1 = 3.2 PP, respectively. PP, percentage points.

Compared with RT-DETR-r18, the proposed model shows smaller attenuation under heavy uneven illumination and heavy motion blur, suggesting better robustness when image quality is degraded but defect regions are not completely occluded. However, under some light disturbance conditions, the attenuation of OURS is slightly larger than that of the baseline. Therefore, the robustness advantage of the proposed model is mainly reflected in its higher retained detection accuracy and better performance under severe illumination variation and motion blur, while heavy dust-like occlusion remains a challenging condition for both models.

## 4 Discussion

This study proposes targeted improvements to RT-DETR-r18 to address several key challenges in shawo radish surface defect detection, including missed detections of small defects, insufficient multi-scale feature representation, and limited feature fusion efficiency. Experimental results show that the proposed model outperforms the baseline RT-DETR-r18 in Precision, Recall, and mAP@50, while further reducing parameter count and computational complexity. These results indicate that the constructed frequency–spatial collaborative modeling framework can effectively improve overall detection performance while maintaining real-time inference capability.

Compared with the YOLO series, the proposed model achieves higher detection accuracy, although its inference speed is lower than that of YOLOv5m, YOLOv8m, YOLOv11m, and YOLOv26m. This suggests that YOLO-based models retain advantages in inference efficiency, but their feature representation ability remains limited when handling shawo radish defects with small scales, weak textures, irregular morphologies, and high inter-class similarity. Faster R-CNN obtains relatively lower Precision and mAP@50, indicating that two-stage detection frameworks may be more susceptible to interference from complex surface textures and background regions in this task. DINO and Deformable DETR achieve better global modeling capability than conventional CNN-based detectors, but their parameter counts and computational costs are considerably higher. Moreover, their performance on crack defects remains limited, suggesting that global or deformable attention alone is insufficient to fully capture slender, low-contrast, and weak-edge defect structures. In contrast, the proposed model achieves a better balance among detection accuracy, model complexity, and inference efficiency, demonstrating its suitability for shawo radish surface defect detection.

From a category-wise perspective, each proposed module contributes differently to different defect types. WT_Block introduces the two-dimensional Haar wavelet transform to achieve multi-resolution feature decomposition and frequency-domain enhancement. Experimental results show that the AP values of mechanical damage, growth nodule, and disease spot increase after introducing this module, with disease spot showing the most evident improvement. This indicates that frequency-domain feature modeling strengthens the model’s ability to characterize texture variations, edge structures, and scale differences. Since disease spot and growth nodule usually contain relatively rich local texture information, while mechanical damage is often accompanied by distinct edge variations, these results are consistent with the design objective of WT_Block.

The AIFI-HiLo module achieves the highest overall mAP@50 among the single-module ablation models. Category-level results show that this module provides particularly notable improvements for disease spot and mechanical damage, while the gain for crack is relatively limited. A possible explanation is that the HiLo attention mechanism models high-frequency local details and low-frequency global semantic information through two separate branches, thereby enhancing the model’s focus on critical defect regions and suppressing background interference. This mechanism is especially beneficial for defects with relatively clear regional characteristics, such as disease spot and mechanical damage. However, for slender and continuous crack defects, attention modeling alone may still be insufficient to fully preserve continuous edge structures, resulting in limited improvement for this category.

VP_CCFM mainly functions in the feature fusion stage. Experimental results show that this module improves cross-scale feature interaction with relatively low computational overhead and achieves the best AP for the crack category among the single-module variants. This suggests that the enhanced feature fusion strategy can better aggregate structural information from different scales and improve the representation of defects with large morphological variations. Meanwhile, the relatively limited improvement for growth nodule indicates that feature fusion alone cannot fully compensate for insufficient local texture representation in complex and weak-textured defects.

Further analysis of the integrated model shows that the proposed model achieves the highest overall mAP@50 and maintains relatively balanced detection performance across the four defect categories. The AP of disease spot reaches 92.7%, representing the largest improvement over the baseline. The AP values of growth nodule and mechanical damage also increase from 81.1 to 81.9% and from 83.0 to 83.9%, respectively. Although the AP improvement for crack remains limited and even shows a slight decrease compared with the baseline in the category-wise AP analysis, the proposed model still improves the overall detection performance. This indicates that crack detection remains challenging due to its slender shape, weak texture features, and low contrast against the radish surface. Overall, WT_Block, AIFI-HiLo, and VP_CCFM form a complementary system for frequency-domain representation, frequency-aware attention modeling, and cross-scale feature fusion. WT_Block provides multi-resolution frequency information, AIFI-HiLo enhances the representation of critical defect regions, and VP_CCFM promotes cross-scale feature transmission. Their synergistic effect ultimately improves the comprehensive detection capability for complex radish surface defects.

It should be noted that, although the proposed model improves the overall detection performance, its enhancement for crack defects remains relatively limited. Crack defects are usually slender and continuous, with low contrast and weak texture responses, and their detection heavily depends on fine-grained edge continuity and local high-frequency feature responses. In this case, excessive global contextual aggregation may introduce a smoothing effect into the feature representation, thereby weakening the discriminability between subtle crack boundaries and the surrounding radish surface. Although the high-frequency branch in AIFI-HiLo is designed to retain local detail cues, the final feature representation is still influenced by the interaction between high-frequency local responses and low-frequency global semantic information. When global semantic aggregation becomes dominant, the weak and elongated responses associated with crack defects may be partially diluted, which may explain the decrease in AP for the crack category. The fact that the VP_CCFM-only variant achieves the highest AP for crack further supports this interpretation. VP_CCFM strengthens cross-scale feature fusion and helps preserve contour continuity across different feature levels, which is particularly important for detecting slender crack structures. Strictly speaking, crack defects can be regarded as thin-structure defects with narrow-width characteristics. For this type of defect, future optimization can draw on small-object and slender-target detection strategies reported in related crop detection studies. For example, a shallow P2 feature layer can be introduced into the backbone or neck to increase the spatial resolution of the detection head, thereby preserving more fine-grained local details. In addition, dynamic upsampling operators can be embedded in the feature fusion stage to adaptively recover spatial details and boundary information, which may reduce the loss of subtle crack features caused by repeated downsampling. Therefore, future work will focus on crack-specific optimization strategies, including shallow feature enhancement, dynamic upsampling (36), edge-preserving constraints, and thin-structure-sensitive detection heads, to further improve the detection accuracy of slender and continuous crack defects.

It should also be noted that the uniform-background setting used in this study may not fully represent real conveyor-belt sorting scenarios. In practical sorting lines, radishes may overlap or contact each other, resulting in contact boundaries, shadows, and local occlusion edges. These visual patterns may resemble slender cracks or mechanical damage and thus increase the risk of background false positives. Meanwhile, partial occlusion of actual defect regions may weaken defect-related texture and boundary cues, leading to missed detections, especially for small nodules, weak disease spots, and thin cracks. Although engineering measures such as single-row feeding, spacing control, and closed light-shielding boxes can reduce these risks, further validation using real conveyor-belt data is still necessary. Future work will collect overlapping and cluttered-background samples and explore occlusion augmentation, domain adaptation, and overlap-aware detection strategies to improve practical robustness.

In addition, although the proposed model achieves an inference speed of 105 FPS, its practical deployment in real sorting lines also depends on conveyor belt speed, camera triggering frequency, actuator response latency, and system integration conditions. Therefore, industrial verification of the proposed algorithm under real sorting scenarios will be carried out in future research.

## 5 Conclusion

This study proposed an improved RT-DETR-r18 framework for shawo radish surface defect detection. By incorporating WT_Block, AIFI-HiLo, and VP_CCFM, the proposed method enhanced multi-scale feature representation, frequency-aware feature modeling, and feature fusion efficiency, thereby addressing the challenges of dense small defects and complex defect patterns.

Experimental results demonstrated that the proposed model achieved a Precision of 94.5%, a Recall of 81.2%, and an mAP@50 of 85.3%, representing improvements of 2.9, 2.8, and 1.9 percentage points over the baseline RT-DETR-r18 model, respectively. Meanwhile, the parameter count and computational complexity were reduced by 7 and 12.7%, respectively, while maintaining real-time inference at 105 FPS. These results indicated that the proposed method achieved an effective balance between detection accuracy and computational efficiency.

Overall, the proposed frequency–spatial collaborative framework provided an effective solution for shawo radish surface defect detection and offered a useful reference for intelligent quality inspection of root vegetable products. Future work will focus on validating the proposed method under broader application scenarios and further improving its deployment adaptability in practical agricultural environments.

## Data Availability

The raw data supporting the conclusions of this article will be made available by the authors, without undue reservation.

## Supplementary material

SUPPLEMENTARY FIGURE 1 Grouped dot plot of per-class AP@50 values for different ablation models.

SUPPLEMENTARY FIGURE 2 Grad-CAM visualization results of different module configurations on representative Shawo radish surface defect samples. Columns A–D correspond to Growth Nodule, Disease Spot, Crack, and Mechanical Damage, respectively. Red boxes indicate annotated defect regions.

SUPPLEMENTARY FIGURE 3 Examples of original and simulated disturbed images used for robustness evaluation. **(A)** Original image. **(B)** Light uneven illumination. **(C)** Heavy uneven illumination. **(D)** Light dust-like occlusion. **(E)** Heavy dust-like occlusion. **(F)** Light motion blur. **(G)** Heavy motion blur.

SUPPLEMENTARY FIGURE 4 mAP@50 comparison under different disturbance conditions.
